# Supplementary material for: A Double Negative Feedback Loop between mTORC1 and AMPK Kinases Guarantees Precise Autophagy Induction upon Cellular Stress
Source: Int J Mol Sci. 2019 Nov 7;20(22):5543. doi: 10.3390/ijms20225543 (PMC6888297; doi:10.3390/ijms20225543)
Supplement: Supplementary file 1 [file ijms-20-05543-s001.zip › ijms-626568-supplementary.docx]

**Supplementary Information**

**“A double negative feedback loop between mTORC1 and AMPK kinases**

**guarantees a precise autophagic response upon cellular stress”**

**I. Mathematical codes for computational simulations**

A biological regulatory network can be translated into a set of ordinary differential equation (ODE) to describe how the concentration/activity of each control element in the network changes with the time. A generic differential equation depicting the temporal changes of a regulatory element is composed of two parts: production and consumption terms. In a cellular protein-protein regulatory network the production can be given by protein synthesis (i.e. transcription and translation) and/or an activation (i.e. post-translational modification) term, while the consumption can be given by protein degradation and/or inactivation term. Usually synthesis and degradation reactions are described by mass action kinetics, whereas protein activity can be described either by mass action or Michaelis-Menten kinetics. Solving a set of non-linear ODEs gives the time evolution of the relative protein concentration/activity (time courses).

The temporal profiles were computed numerically using *XPP-AUT*. All the simulations presented in the text are based on the following XPP codes which contains ODEs. The rate constants (k) have the dimension of min^-1^ and Michaelis constants (*J*) are dimensionless. The proteins levels/activities are given in arbitrary units (a.u).

*The code for simulating time series when mTOR ┤AMPK connection is not present*

# a model to simulate mTOR-AMPK-ULK1 regulatory triangle with XPP-AUT

# the code for simulating time series when mTOR ┤AMPK connection is not present

# initial conditions

init ULK1=0.0055, AMPK=0.1017, mTOR=0.4691, ATG=0.0214, starv=0, res=0, rap=0, cc=0

# differential equations

# ULK1 represents the active form of ULK1

ULK1' = (kaulk + kaulk"*delay(AMPK,tau1))*(Ulk1T-ULK1)/(Julk + Ulk1T-ULK1) - (kiulk + kiulk'*mTOR)*ULK1/(Julk + ULK1)

# AMPK represents the active, phosphorlyated form of AMPK

AMPK' = (kaak + kaak*starv + kaak*res)*(AMPKT-AMPK)/(Jampk + AMPKT-AMPK) - (kiak + kiak*cc + kiak'*ULK1)*AMPK/(Jampk + AMPK)

# mTOR represents the active form of mTORC1 complex

mTOR' = kamtor*(mTORT-mTOR)/(Jmtor+mTORT-mTOR) - (kimtor + kimtor'*AMPK + kimtor"*ULK1 + kimtor*res + kimtor*rap)*mTOR/(Jmtor+mTOR)

# ATG represents the active ATG genes during autophagy; when ATG is active we assumes that autophagy is also active

ATG' = (kaau + kaau'*ULK1)*(1-ATG) - (kiau + kiau'*mTOR)*ATG

# starv represents the increasing ratio of AMP/ATP during starvation

starv' = kistv*(starvT-starv)/(Jstv + starvT-starv) - kostv*starv/(Jstv+starv)

# res represents the resveratrol up-taken by the cell

res' = kires*(resT-res)/(Jres + resT-res) - kores*res/(Jres+res)

# rap represents the rapamycin up-taken by the cell

rap' = kirap*(rapT-rap)/(Jrap + rapT-rap) - korap*rap/(Jrap+rap)

# cc represents the Compound C up-taken by the cell

cc' = kicc*(ccT-cc)/(Jcc + ccT-cc) - kocc*cc/(Jcc+cc)

# parameters

# simulating starvation: starvT=2

# simulating rapamycin treatment: rapT=2.25

# simulating resveratrol treatment: resT=1.5

# simulating ULK1 silencing: ULK1T=0.001

# simulating TSC1/TSC2 silencing: kamtor=0.05

# simulating Compound C treatment: CCT=100

p kaulk=5, kaulk"=1, kiulk=0.1, kiulk'=30, julk=0.01, Ulk1T=1

p kaak=0.35, kiak=0.5, kiak'=150, AMPKT=1, Jampk=0.5

p kamtor=0.025, kimtor=0.0075, kimtor'=0.15, kimtor"=0.5, Jmtor=0.1, mTORT=1

p kaau=0.01, kaau'=3, kiau=0.75, kiau'=1

p starvT=0, kistv=0.105, Jstv=0.03, kostv=0.1

p resT=0, kires=0.0025, Jres=0.75, kores=0.00075

p rapT=0, kirap=0.125, Jrap=0.1, korap=0.1

p ccT=0, kicc=0.5, Jcc=0.1, kocc=0.1

p tau1=5

# numerics

@ TOTAL=1, METH=stiff, delay=50

done

*The code for simulating time series when mTOR ┤AMPK connection is present*

# a model to simulate mTOR-AMPK-ULK1 regulatory triangle with XPP-AUT

# the code for simulating time series when mTOR ┤AMPK connection is present

# initial conditions

init ULK1=0.0008, AMPK=0.0161, mTOR=0.7013, ATG=0.0568, starv=0, res=0, rap=0, cc=0

# differential equations

# ULK1 represents the active form of ULK1

ULK1' = (kaulk + kaulk"*delay(AMPK,tau1))*(Ulk1T-ULK1)/(Julk + Ulk1T-ULK1) - (kiulk + kiulk'*mTOR)*ULK1/(Julk + ULK1)

# AMPK represents the active, phosphorlyated form of AMPK

AMPK' = (kaak + kaak*starv + kaak*res)*(AMPKT-AMPK)/(Jampk + AMPKT-AMPK) - (kiak + kiak*cc + kiak'*ULK1 + kiak"*mTOR)*AMPK/(Jampk + AMPK)

# mTOR represents the active form of mTORC1 complex

mTOR' = kamtor*(mTORT-mTOR)/(Jmtor+mTORT-mTOR) - (kimtor + kimtor'*AMPK + kimtor"*ULK1 + kimtor*res + kimtor*rap)*mTOR/(Jmtor+mTOR)

# ATG represents the active ATG genes during autophagy; when ATG is active we assumes that autophagy is also active

ATG' = (kaau + kaau'*ULK1)*(1-ATG) - (kiau + kiau'*mTOR)*ATG

# starv represents the increasing ratio of AMP/ATP during starvation

starv' = kistv*(starvT-starv)/(Jstv + starvT-starv) - kostv*starv/(Jstv+starv)

# res represents the resveratrol up-taken by the cell

res' = kires*(resT-res)/(Jres + resT-res) - kores*res/(Jres+res)

# rap represents the rapamycin up-taken by the cell

rap' = kirap*(rapT-rap)/(Jrap + rapT-rap) - korap*rap/(Jrap+rap)

# cc represents the Compound C up-taken by the cell

cc' = kicc*(ccT-cc)/(Jcc + ccT-cc) - kocc*cc/(Jcc+cc)

# parameters

# simulating starvation: starvT=0.75

# simulating rapamycin treatment: rapT=10

# simulating resveratrol treatment: resT=0.175

# simulating ULK1 silencing: ULK1T=0.001

# simulating TSC1/TSC2 silencing: kamtor=0.05

# simulating Compound C treatment: CCT=100

p kaulk=0.001, kaulk"=3, kiulk=0.1, kiulk'=0.75, julk=0.01, Ulk1T=1

p kaak=0.5, kiak=0.1, kiak'=1.5, kiak"=15, AMPKT=1, Jampk=0.5

p kamtor=0.015, kimtor=0.01, kimtor'=0.15, kimtor"=0.5, Jmtor=0.1, mTORT=1

p kaau=0.1, kaau'=3, kiau=1, kiau'=1

p starvT=0, kistv=0.1, Jstv=0.1, kostv=0.1

p rapT=0, kirap=0.15, Jrap=0.1, korap=0.1

p ccT=0, kicc=1, Jcc=0.5, kocc=0.001

p tau1=50

# numerics

@ TOTAL=1, METH=stiff, delay=50

done

**II. Figures**

1. **(B)**


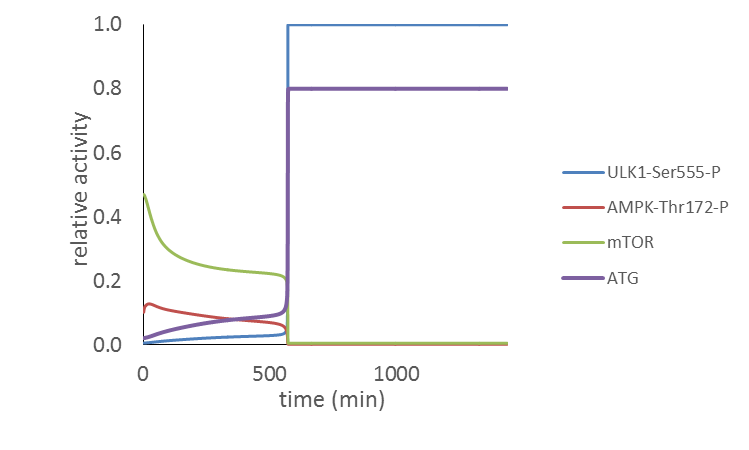

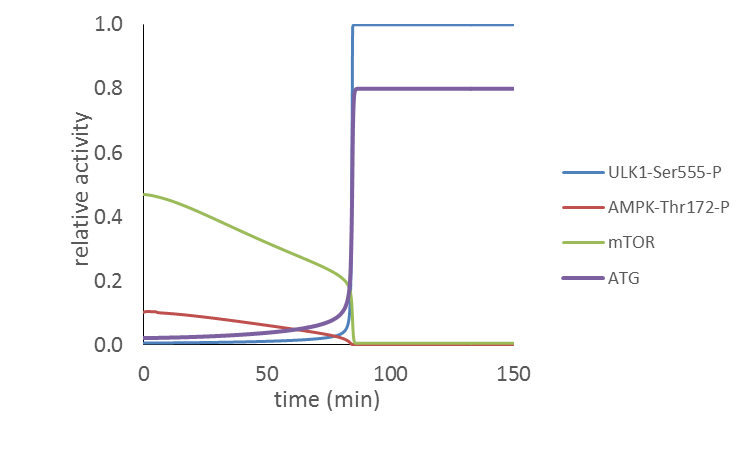


**(C) (D)**


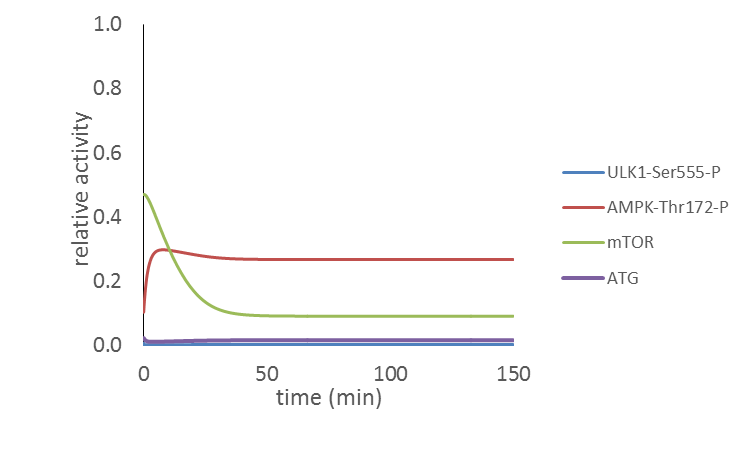

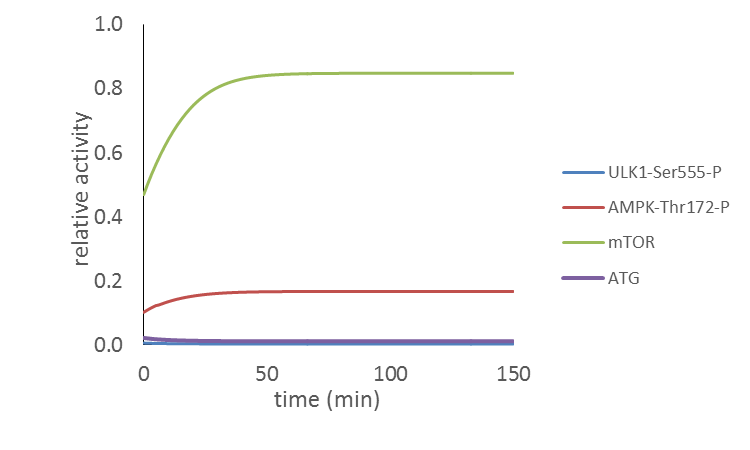


**Supplementary Figure 1.** Computer simulation of **(A)** starvation (starvT=2); **(B)** rapamycin treatment (rapt=2.25); **(C)** ULK1 silencing (ULK1T=0.001) and **(D)** mTOR hyperactivation with siTSC1/TSC2 (kamtor=0.05) when mTOR ┤AMPK connection is not present in the regulatory network. The relative activity of AMPK-Thr172-P, mTOR, ULK1-Ser555-P and autophagy (ATG) are plotted in time.

**(A) (B)**


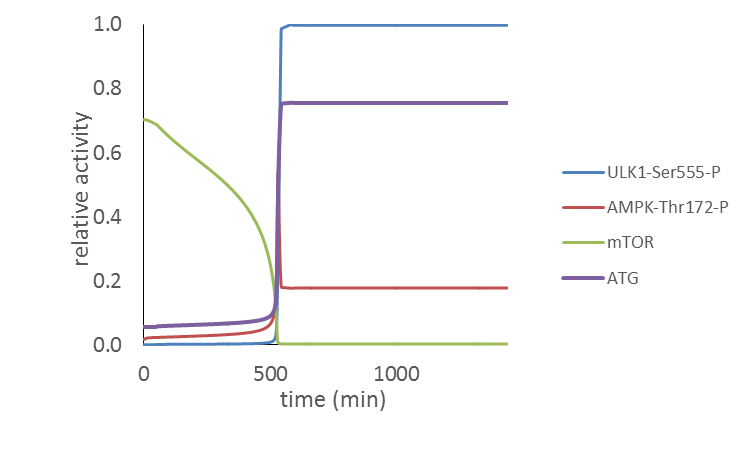

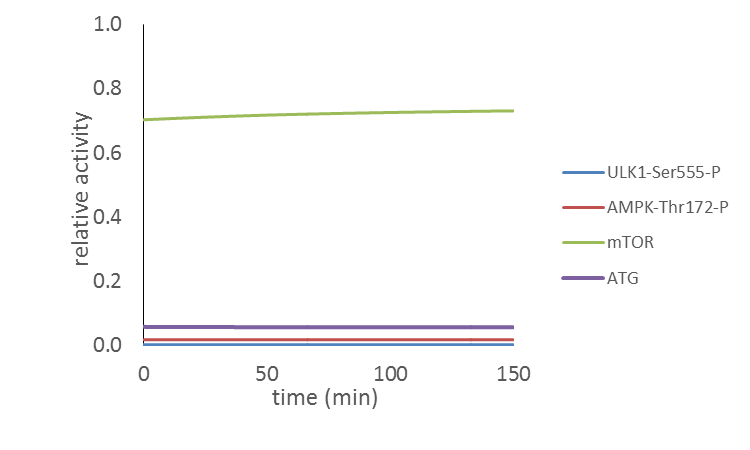


**(C) (D)**


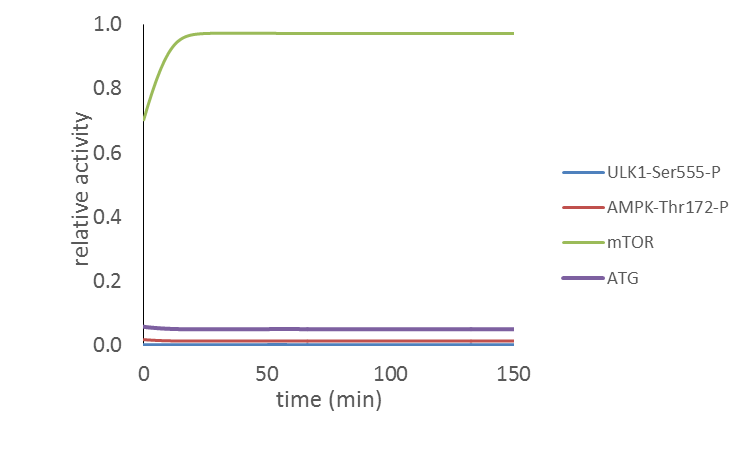

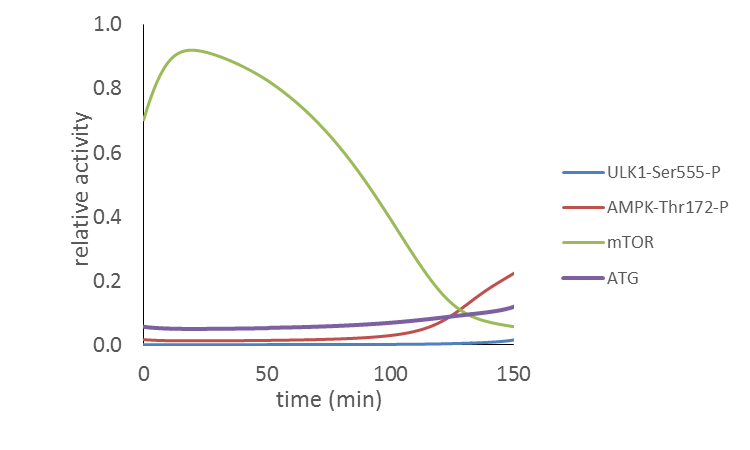


**(E) (F)**


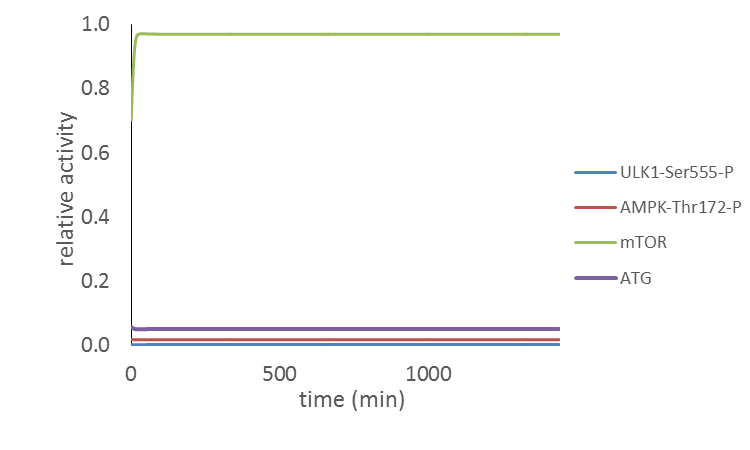

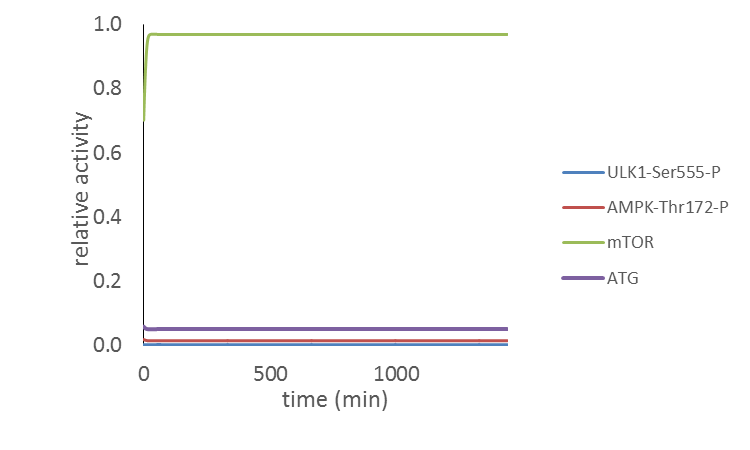


**Supplementary Figure 2.** Computer simulation of AMPK-mTOR-ULK1 regulatory triangle when mTOR ┤AMPK connection is present in the control network. Time series data are plotted mimicking **(A)** starvation (starvT=0.75); **(B)** ULK1 silencing (ULK1T=0.001); **(C)** mTOR hyperactivation with siTSC1/TSC2 (kamtor=0.05); and combined treatment of TSC1/2 silencing **(D)** + addition of rapamycin (kamtor=0.05, rapT=10); **(E)** + starvation (kamtor=0.05, starvT=0.75) or **(F)** + addition of resveratrol (kamtor=0.05, resT=0.175). The relative activity of AMPK-Thr172-P, mTOR, ULK1-Ser555-P and autophagy (ATG) are plotted in time.

**III. Tables**

**Supplementary Table 1. Collecting data from literature about activity changes of AMPK, mTOR and ULK1 after various treatments.** ATG means active autophagy.

**Supplementary Table 2. Comparison of the two mathematical models i.e. mTOR inhibits AMPK directly or not by simulating various treatments.** The activity change of AMPK, mTOR and ULK1 is presented after the treatments. ATG means active autophagy. Red background refers that this computational result is not matched the data found in the literature, while red notes present our predictions since these treatments have not carried out yet experimentally.

**IV. Introducing the theoretical analysis of phosphorylation site search on AMPK**

PhosphoSite Plus (https://www.phosphosite.org/proteinAction?id=564&showAllSites=true) shows the preferred Ser and Thr phosphorylation sites of mTOR kinase:


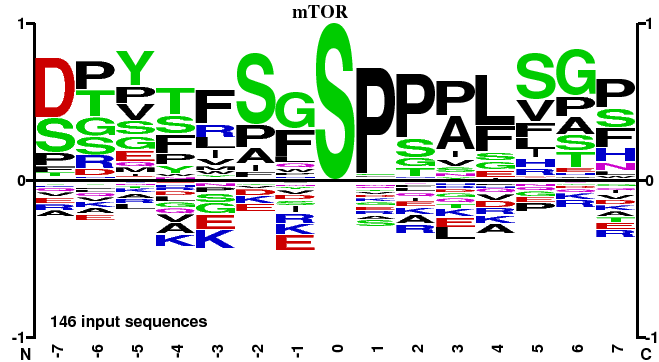

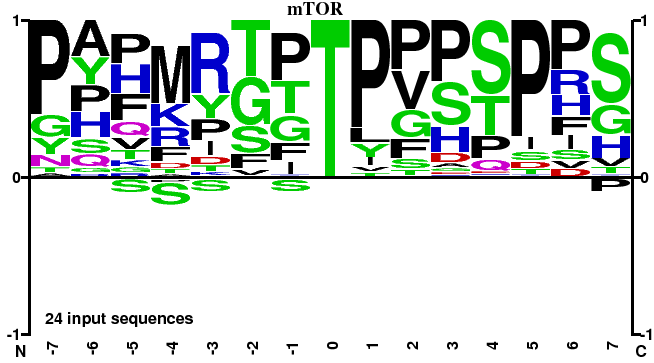


Using NetPhos 3.1. (<http://www.cbs.dtu.dk/services/NetPhos/>) the potential serine and thr phosphorylation sites were searched in human AMPK protein sequence (the algorithm of this freely availably software can be found in Blom et al., 2014). The program identified four phosphorylation sites with the following amino acid sequences:

- Thr-232: DHVPTLFKK
- Ser-356: YLATSPPDS
- Thr-488: AKSGTATPQ
- Ser-496: QRSGSVSNY

where the yellow colour shows that this amino acid around the potential Ser and Thr resiudes (marked with red background) are preferred by mTOR kinase. According to analysis done by NetPhos 3.1., The-488 and Ser-496 residues are really close to each other further suggesting that these two might be regulated similarly by mTOR-dependent phosphorylation.

**V. References**

1. [Biochim Biophys Acta.](https://www.ncbi.nlm.nih.gov/pubmed/26975583) 2016 Jun;1863:1200-7. doi: 10.1016/j.bbamcr.2016.03.009. Rapamycin requires AMPK activity and p27 expression for promoting autophagy-dependent Tsc2-null cell survival. [Campos T](https://www.ncbi.nlm.nih.gov/pubmed/?term=Campos%20T%5BAuthor%5D&cauthor=true&cauthor_uid=26975583), [Ziehe J](https://www.ncbi.nlm.nih.gov/pubmed/?term=Ziehe%20J%5BAuthor%5D&cauthor=true&cauthor_uid=26975583), [Fuentes-Villalobos F](https://www.ncbi.nlm.nih.gov/pubmed/?term=Fuentes-Villalobos%20F%5BAuthor%5D&cauthor=true&cauthor_uid=26975583), [Riquelme O](https://www.ncbi.nlm.nih.gov/pubmed/?term=Riquelme%20O%5BAuthor%5D&cauthor=true&cauthor_uid=26975583), [Peña D](https://www.ncbi.nlm.nih.gov/pubmed/?term=Pe%C3%B1a%20D%5BAuthor%5D&cauthor=true&cauthor_uid=26975583), [Troncoso R](https://www.ncbi.nlm.nih.gov/pubmed/?term=Troncoso%20R%5BAuthor%5D&cauthor=true&cauthor_uid=26975583), [Lavandero S](https://www.ncbi.nlm.nih.gov/pubmed/?term=Lavandero%20S%5BAuthor%5D&cauthor=true&cauthor_uid=26975583), [Morin V](https://www.ncbi.nlm.nih.gov/pubmed/?term=Morin%20V%5BAuthor%5D&cauthor=true&cauthor_uid=26975583), [Pincheira R](https://www.ncbi.nlm.nih.gov/pubmed/?term=Pincheira%20R%5BAuthor%5D&cauthor=true&cauthor_uid=26975583), [Castro AF](https://www.ncbi.nlm.nih.gov/pubmed/?term=Castro%20AF%5BAuthor%5D&cauthor=true&cauthor_uid=26975583).
2. [J Cell Physiol.](https://www.ncbi.nlm.nih.gov/pubmed/30549029) 2018 Dec 13. doi: 10.1002/jcp.27979. Rapamycin enhances growth inhibition on urothelial carcinoma cells through LKB1 deficiency-mediated mitochondrial dysregulation. [Whang YM](https://www.ncbi.nlm.nih.gov/pubmed/?term=Whang%20YM%5BAuthor%5D&cauthor=true&cauthor_uid=30549029), [Kim MJ](https://www.ncbi.nlm.nih.gov/pubmed/?term=Kim%20MJ%5BAuthor%5D&cauthor=true&cauthor_uid=30549029), [Cho MJ](https://www.ncbi.nlm.nih.gov/pubmed/?term=Cho%20MJ%5BAuthor%5D&cauthor=true&cauthor_uid=30549029), [Yoon H](https://www.ncbi.nlm.nih.gov/pubmed/?term=Yoon%20H%5BAuthor%5D&cauthor=true&cauthor_uid=30549029), [Choi YW](https://www.ncbi.nlm.nih.gov/pubmed/?term=Choi%20YW%5BAuthor%5D&cauthor=true&cauthor_uid=30549029), [Kim TH](https://www.ncbi.nlm.nih.gov/pubmed/?term=Kim%20TH%5BAuthor%5D&cauthor=true&cauthor_uid=30549029), [Chang IH](https://www.ncbi.nlm.nih.gov/pubmed/?term=Chang%20IH%5BAuthor%5D&cauthor=true&cauthor_uid=30549029).
3. [Diabetes.](https://www.ncbi.nlm.nih.gov/pubmed/16873680) 2006 Aug;55(8):2180-91. Polyphenols stimulate AMP-activated protein kinase, lower lipids, and inhibit accelerated atherosclerosis in diabetic LDL receptor-deficient mice. [Zang M](https://www.ncbi.nlm.nih.gov/pubmed/?term=Zang%20M%5BAuthor%5D&cauthor=true&cauthor_uid=16873680), [Xu S](https://www.ncbi.nlm.nih.gov/pubmed/?term=Xu%20S%5BAuthor%5D&cauthor=true&cauthor_uid=16873680), [Maitland-Toolan KA](https://www.ncbi.nlm.nih.gov/pubmed/?term=Maitland-Toolan%20KA%5BAuthor%5D&cauthor=true&cauthor_uid=16873680), [Zuccollo A](https://www.ncbi.nlm.nih.gov/pubmed/?term=Zuccollo%20A%5BAuthor%5D&cauthor=true&cauthor_uid=16873680), [Hou X](https://www.ncbi.nlm.nih.gov/pubmed/?term=Hou%20X%5BAuthor%5D&cauthor=true&cauthor_uid=16873680), [Jiang B](https://www.ncbi.nlm.nih.gov/pubmed/?term=Jiang%20B%5BAuthor%5D&cauthor=true&cauthor_uid=16873680), [Wierzbicki M](https://www.ncbi.nlm.nih.gov/pubmed/?term=Wierzbicki%20M%5BAuthor%5D&cauthor=true&cauthor_uid=16873680), [Verbeuren TJ](https://www.ncbi.nlm.nih.gov/pubmed/?term=Verbeuren%20TJ%5BAuthor%5D&cauthor=true&cauthor_uid=16873680), [Cohen RA](https://www.ncbi.nlm.nih.gov/pubmed/?term=Cohen%20RA%5BAuthor%5D&cauthor=true&cauthor_uid=16873680).
4. [BMC Cancer](https://www.ncbi.nlm.nih.gov/pmc/articles/PMC5964919/). 2018; 18: 581. doi: [10.1186/s12885-018-4504-5](https://dx.doi.org/10.1186%2Fs12885-018-4504-5). Resveratrol induces autophagy-dependent apoptosis in HL-60 cells. [Yingying Fan](https://www.ncbi.nlm.nih.gov/pubmed/?term=Fan%20Y%5BAuthor%5D&cauthor=true&cauthor_uid=29788929), [Jen-Fu Chiu](https://www.ncbi.nlm.nih.gov/pubmed/?term=Chiu%20JF%5BAuthor%5D&cauthor=true&cauthor_uid=29788929), [Jing Liu](https://www.ncbi.nlm.nih.gov/pubmed/?term=Liu%20J%5BAuthor%5D&cauthor=true&cauthor_uid=29788929), [Yan Deng](https://www.ncbi.nlm.nih.gov/pubmed/?term=Deng%20Y%5BAuthor%5D&cauthor=true&cauthor_uid=29788929), [Cheng Xu](https://www.ncbi.nlm.nih.gov/pubmed/?term=Xu%20C%5BAuthor%5D&cauthor=true&cauthor_uid=29788929), [Jun Zhang](https://www.ncbi.nlm.nih.gov/pubmed/?term=Zhang%20J%5BAuthor%5D&cauthor=true&cauthor_uid=29788929), and [Guanwu Li](https://www.ncbi.nlm.nih.gov/pubmed/?term=Li%20G%5BAuthor%5D&cauthor=true&cauthor_uid=29788929)
5. [Int Immunopharmacol.](https://www.ncbi.nlm.nih.gov/pubmed/26774212) 2016 Mar;32:1-7. doi: 10.1016/j.intimp.2016.01.002. Resveratrol-induced autophagy promotes survival and attenuates doxorubicin-induced cardiotoxicity. [Gu J](https://www.ncbi.nlm.nih.gov/pubmed/?term=Gu%20J%5BAuthor%5D&cauthor=true&cauthor_uid=26774212), [Hu W](https://www.ncbi.nlm.nih.gov/pubmed/?term=Hu%20W%5BAuthor%5D&cauthor=true&cauthor_uid=26774212), [Song ZP](https://www.ncbi.nlm.nih.gov/pubmed/?term=Song%20ZP%5BAuthor%5D&cauthor=true&cauthor_uid=26774212), [Chen YG](https://www.ncbi.nlm.nih.gov/pubmed/?term=Chen%20YG%5BAuthor%5D&cauthor=true&cauthor_uid=26774212), [Zhang DD](https://www.ncbi.nlm.nih.gov/pubmed/?term=Zhang%20DD%5BAuthor%5D&cauthor=true&cauthor_uid=26774212), [Wang CQ](https://www.ncbi.nlm.nih.gov/pubmed/?term=Wang%20CQ%5BAuthor%5D&cauthor=true&cauthor_uid=26774212)
6. [Gynecol Oncol.](https://www.ncbi.nlm.nih.gov/pubmed/17825886) 2007 Dec;107(3):450-7. Resveratrol inhibits glucose metabolism in human ovarian cancer cells. [Kueck A](https://www.ncbi.nlm.nih.gov/pubmed/?term=Kueck%20A%5BAuthor%5D&cauthor=true&cauthor_uid=17825886), [Opipari AW Jr](https://www.ncbi.nlm.nih.gov/pubmed/?term=Opipari%20AW%20Jr%5BAuthor%5D&cauthor=true&cauthor_uid=17825886), [Griffith KA](https://www.ncbi.nlm.nih.gov/pubmed/?term=Griffith%20KA%5BAuthor%5D&cauthor=true&cauthor_uid=17825886), [Tan L](https://www.ncbi.nlm.nih.gov/pubmed/?term=Tan%20L%5BAuthor%5D&cauthor=true&cauthor_uid=17825886), [Choi M](https://www.ncbi.nlm.nih.gov/pubmed/?term=Choi%20M%5BAuthor%5D&cauthor=true&cauthor_uid=17825886), [Huang J](https://www.ncbi.nlm.nih.gov/pubmed/?term=Huang%20J%5BAuthor%5D&cauthor=true&cauthor_uid=17825886), [Wahl H](https://www.ncbi.nlm.nih.gov/pubmed/?term=Wahl%20H%5BAuthor%5D&cauthor=true&cauthor_uid=17825886), [Liu JR](https://www.ncbi.nlm.nih.gov/pubmed/?term=Liu%20JR%5BAuthor%5D&cauthor=true&cauthor_uid=17825886).
7. [Diabetes.](https://www.ncbi.nlm.nih.gov/pubmed/16873680%20) 2006 Aug;55(8):2180-91. Polyphenols stimulate AMP-activated protein kinase, lower lipids, and inhibit accelerated atherosclerosis in diabetic LDL receptor-deficient mice. [Zang M](https://www.ncbi.nlm.nih.gov/pubmed/?term=Zang%20M%5BAuthor%5D&cauthor=true&cauthor_uid=16873680), [Xu S](https://www.ncbi.nlm.nih.gov/pubmed/?term=Xu%20S%5BAuthor%5D&cauthor=true&cauthor_uid=16873680), [Maitland-Toolan KA](https://www.ncbi.nlm.nih.gov/pubmed/?term=Maitland-Toolan%20KA%5BAuthor%5D&cauthor=true&cauthor_uid=16873680), [Zuccollo A](https://www.ncbi.nlm.nih.gov/pubmed/?term=Zuccollo%20A%5BAuthor%5D&cauthor=true&cauthor_uid=16873680), [Hou X](https://www.ncbi.nlm.nih.gov/pubmed/?term=Hou%20X%5BAuthor%5D&cauthor=true&cauthor_uid=16873680), [Jiang B](https://www.ncbi.nlm.nih.gov/pubmed/?term=Jiang%20B%5BAuthor%5D&cauthor=true&cauthor_uid=16873680), [Wierzbicki M](https://www.ncbi.nlm.nih.gov/pubmed/?term=Wierzbicki%20M%5BAuthor%5D&cauthor=true&cauthor_uid=16873680), [Verbeuren TJ](https://www.ncbi.nlm.nih.gov/pubmed/?term=Verbeuren%20TJ%5BAuthor%5D&cauthor=true&cauthor_uid=16873680), [Cohen RA](https://www.ncbi.nlm.nih.gov/pubmed/?term=Cohen%20RA%5BAuthor%5D&cauthor=true&cauthor_uid=16873680).
8. [Sci Rep](https://www.ncbi.nlm.nih.gov/pmc/articles/PMC4763238/). 2016; 6: 21772. doi: [10.1038/srep21772](https://dx.doi.org/10.1038%2Fsrep21772). Resveratrol induces autophagy by directly inhibiting mTOR through ATP competition. [Dohyun Park](https://www.ncbi.nlm.nih.gov/pubmed/?term=Park%20D%5BAuthor%5D&cauthor=true&cauthor_uid=26902888), [Heeyoon Jeong](https://www.ncbi.nlm.nih.gov/pubmed/?term=Jeong%20H%5BAuthor%5D&cauthor=true&cauthor_uid=26902888), [Mi Nam Lee](https://www.ncbi.nlm.nih.gov/pubmed/?term=Lee%20MN%5BAuthor%5D&cauthor=true&cauthor_uid=26902888), [Ara Koh](https://www.ncbi.nlm.nih.gov/pubmed/?term=Koh%20A%5BAuthor%5D&cauthor=true&cauthor_uid=26902888), [Ohman Kwon](https://www.ncbi.nlm.nih.gov/pubmed/?term=Kwon%20O%5BAuthor%5D&cauthor=true&cauthor_uid=26902888), [Yong Ryoul Yang](https://www.ncbi.nlm.nih.gov/pubmed/?term=Yang%20YR%5BAuthor%5D&cauthor=true&cauthor_uid=26902888), [Jungeun Noh](https://www.ncbi.nlm.nih.gov/pubmed/?term=Noh%20J%5BAuthor%5D&cauthor=true&cauthor_uid=26902888), [Pann-Ghill Suh](https://www.ncbi.nlm.nih.gov/pubmed/?term=Suh%20PG%5BAuthor%5D&cauthor=true&cauthor_uid=26902888), [Hwangseo Park](https://www.ncbi.nlm.nih.gov/pubmed/?term=Park%20H%5BAuthor%5D&cauthor=true&cauthor_uid=26902888), and [Sung Ho Ryu](https://www.ncbi.nlm.nih.gov/pubmed/?term=Ryu%20SH%5BAuthor%5D&cauthor=true&cauthor_uid=26902888)
9. [Biochem Biophys Res Commun.](https://www.ncbi.nlm.nih.gov/pubmed/25117440) 2014 Sep 26;452(3):308-14. doi: 10.1016/j.bbrc.2014.08.017. Ambra1 modulates starvation-induced autophagy through AMPK signaling pathway in cardiomyocytes. [Shi C](https://www.ncbi.nlm.nih.gov/pubmed/?term=Shi%20C%5BAuthor%5D&cauthor=true&cauthor_uid=25117440), [Wu J](https://www.ncbi.nlm.nih.gov/pubmed/?term=Wu%20J%5BAuthor%5D&cauthor=true&cauthor_uid=25117440), [Fu M](https://www.ncbi.nlm.nih.gov/pubmed/?term=Fu%20M%5BAuthor%5D&cauthor=true&cauthor_uid=25117440), [Zhang B](https://www.ncbi.nlm.nih.gov/pubmed/?term=Zhang%20B%5BAuthor%5D&cauthor=true&cauthor_uid=25117440), [Wang J](https://www.ncbi.nlm.nih.gov/pubmed/?term=Wang%20J%5BAuthor%5D&cauthor=true&cauthor_uid=25117440), [Yang X](https://www.ncbi.nlm.nih.gov/pubmed/?term=Yang%20X%5BAuthor%5D&cauthor=true&cauthor_uid=25117440), [Chi Y](https://www.ncbi.nlm.nih.gov/pubmed/?term=Chi%20Y%5BAuthor%5D&cauthor=true&cauthor_uid=25117440).
10. [Biochem Biophys Res Commun.](https://www.ncbi.nlm.nih.gov/pubmed/29284117) 2018 Jan 22;495(4):2566-2572. doi: 10.1016/j.bbrc.2017.12.141. Knockdown of AMPKα decreases ATM expression and increases radiosensitivity under hypoxia and nutrient starvation in an SV40-transformed human fibroblast cell line, LM217. [Murata Y](https://www.ncbi.nlm.nih.gov/pubmed/?term=Murata%20Y%5BAuthor%5D&cauthor=true&cauthor_uid=29284117), [Hashimoto T](https://www.ncbi.nlm.nih.gov/pubmed/?term=Hashimoto%20T%5BAuthor%5D&cauthor=true&cauthor_uid=29284117), [Urushihara Y](https://www.ncbi.nlm.nih.gov/pubmed/?term=Urushihara%20Y%5BAuthor%5D&cauthor=true&cauthor_uid=29284117), [Shiga S](https://www.ncbi.nlm.nih.gov/pubmed/?term=Shiga%20S%5BAuthor%5D&cauthor=true&cauthor_uid=29284117), [Takeda K](https://www.ncbi.nlm.nih.gov/pubmed/?term=Takeda%20K%5BAuthor%5D&cauthor=true&cauthor_uid=29284117), [Jingu K](https://www.ncbi.nlm.nih.gov/pubmed/?term=Jingu%20K%5BAuthor%5D&cauthor=true&cauthor_uid=29284117), [Hosoi Y](https://www.ncbi.nlm.nih.gov/pubmed/?term=Hosoi%20Y%5BAuthor%5D&cauthor=true&cauthor_uid=29284117).
11. [Cell Rep.](https://www.ncbi.nlm.nih.gov/pubmed/30517873) 2018 Dec 4;25(10):2878-2890.e4. doi: 10.1016/j.celrep.2018.11.042. ULK1 O-GlcNAcylation Is Crucial for Activating VPS34 via ATG14L during Autophagy Initiation. [Pyo KE](https://www.ncbi.nlm.nih.gov/pubmed/?term=Pyo%20KE%5BAuthor%5D&cauthor=true&cauthor_uid=30517873), [Kim CR](https://www.ncbi.nlm.nih.gov/pubmed/?term=Kim%20CR%5BAuthor%5D&cauthor=true&cauthor_uid=30517873), [Lee M](https://www.ncbi.nlm.nih.gov/pubmed/?term=Lee%20M%5BAuthor%5D&cauthor=true&cauthor_uid=30517873), [Kim JS](https://www.ncbi.nlm.nih.gov/pubmed/?term=Kim%20JS%5BAuthor%5D&cauthor=true&cauthor_uid=30517873), [Kim KI](https://www.ncbi.nlm.nih.gov/pubmed/?term=Kim%20KI%5BAuthor%5D&cauthor=true&cauthor_uid=30517873), [Baek SH](https://www.ncbi.nlm.nih.gov/pubmed/?term=Baek%20SH%5BAuthor%5D&cauthor=true&cauthor_uid=30517873).
12. [Int J Biochem Cell Biol.](https://www.ncbi.nlm.nih.gov/pubmed/25043686) 2014 Sep;54:208-16. doi: 10.1016/j.biocel.2014.07.008. Post-transcriptional regulation of autophagy in C2C12 myotubes following starvation and nutrient restoration. [Desgeorges MM](https://www.ncbi.nlm.nih.gov/pubmed/?term=Desgeorges%20MM%5BAuthor%5D&cauthor=true&cauthor_uid=25043686), [Freyssenet D](https://www.ncbi.nlm.nih.gov/pubmed/?term=Freyssenet%20D%5BAuthor%5D&cauthor=true&cauthor_uid=25043686), [Chanon S](https://www.ncbi.nlm.nih.gov/pubmed/?term=Chanon%20S%5BAuthor%5D&cauthor=true&cauthor_uid=25043686), [Castells J](https://www.ncbi.nlm.nih.gov/pubmed/?term=Castells%20J%5BAuthor%5D&cauthor=true&cauthor_uid=25043686), [Pugnière P](https://www.ncbi.nlm.nih.gov/pubmed/?term=Pugni%C3%A8re%20P%5BAuthor%5D&cauthor=true&cauthor_uid=25043686), [Béchet D](https://www.ncbi.nlm.nih.gov/pubmed/?term=B%C3%A9chet%20D%5BAuthor%5D&cauthor=true&cauthor_uid=25043686), [Peinnequin A](https://www.ncbi.nlm.nih.gov/pubmed/?term=Peinnequin%20A%5BAuthor%5D&cauthor=true&cauthor_uid=25043686), [Devillard X](https://www.ncbi.nlm.nih.gov/pubmed/?term=Devillard%20X%5BAuthor%5D&cauthor=true&cauthor_uid=25043686), [Defour A](https://www.ncbi.nlm.nih.gov/pubmed/?term=Defour%20A%5BAuthor%5D&cauthor=true&cauthor_uid=25043686).
13. [Nat Cell Biol.](https://www.ncbi.nlm.nih.gov/pubmed/21258367) 2011 Feb;13(2):132-41. doi: 10.1038/ncb2152. AMPK and mTOR regulate autophagy through direct phosphorylation of Ulk1. [Kim J](https://www.ncbi.nlm.nih.gov/pubmed/?term=Kim%20J%5BAuthor%5D&cauthor=true&cauthor_uid=21258367), [Kundu M](https://www.ncbi.nlm.nih.gov/pubmed/?term=Kundu%20M%5BAuthor%5D&cauthor=true&cauthor_uid=21258367), [Viollet B](https://www.ncbi.nlm.nih.gov/pubmed/?term=Viollet%20B%5BAuthor%5D&cauthor=true&cauthor_uid=21258367), [Guan KL](https://www.ncbi.nlm.nih.gov/pubmed/?term=Guan%20KL%5BAuthor%5D&cauthor=true&cauthor_uid=21258367).
14. [Cell Rep.](https://www.ncbi.nlm.nih.gov/pubmed/30517873) 2018 Dec 4;25(10):2878-2890.e4. doi: 10.1016/j.celrep.2018.11.042. ULK1 O-GlcNAcylation Is Crucial for Activating VPS34 via ATG14L during Autophagy Initiation. [Pyo KE](https://www.ncbi.nlm.nih.gov/pubmed/?term=Pyo%20KE%5BAuthor%5D&cauthor=true&cauthor_uid=30517873), [Kim CR](https://www.ncbi.nlm.nih.gov/pubmed/?term=Kim%20CR%5BAuthor%5D&cauthor=true&cauthor_uid=30517873), [Lee M](https://www.ncbi.nlm.nih.gov/pubmed/?term=Lee%20M%5BAuthor%5D&cauthor=true&cauthor_uid=30517873), [Kim JS](https://www.ncbi.nlm.nih.gov/pubmed/?term=Kim%20JS%5BAuthor%5D&cauthor=true&cauthor_uid=30517873), [Kim KI](https://www.ncbi.nlm.nih.gov/pubmed/?term=Kim%20KI%5BAuthor%5D&cauthor=true&cauthor_uid=30517873), [Baek SH](https://www.ncbi.nlm.nih.gov/pubmed/?term=Baek%20SH%5BAuthor%5D&cauthor=true&cauthor_uid=30517873).
15. [Biochem Biophys Res Commun.](https://www.ncbi.nlm.nih.gov/pubmed/29284117) 2018 Jan 22;495(4):2566-2572. doi: 10.1016/j.bbrc.2017.12.141. Knockdown of AMPKα decreases ATM expression and increases radiosensitivity under hypoxia and nutrient starvation in an SV40-transformed human fibroblast cell line, LM217. [Murata Y](https://www.ncbi.nlm.nih.gov/pubmed/?term=Murata%20Y%5BAuthor%5D&cauthor=true&cauthor_uid=29284117), [Hashimoto T](https://www.ncbi.nlm.nih.gov/pubmed/?term=Hashimoto%20T%5BAuthor%5D&cauthor=true&cauthor_uid=29284117), [Urushihara Y](https://www.ncbi.nlm.nih.gov/pubmed/?term=Urushihara%20Y%5BAuthor%5D&cauthor=true&cauthor_uid=29284117), [Shiga S](https://www.ncbi.nlm.nih.gov/pubmed/?term=Shiga%20S%5BAuthor%5D&cauthor=true&cauthor_uid=29284117), [Takeda K](https://www.ncbi.nlm.nih.gov/pubmed/?term=Takeda%20K%5BAuthor%5D&cauthor=true&cauthor_uid=29284117), [Jingu K](https://www.ncbi.nlm.nih.gov/pubmed/?term=Jingu%20K%5BAuthor%5D&cauthor=true&cauthor_uid=29284117), [Hosoi Y](https://www.ncbi.nlm.nih.gov/pubmed/?term=Hosoi%20Y%5BAuthor%5D&cauthor=true&cauthor_uid=29284117).
16. [Cancer Sci](https://www.ncbi.nlm.nih.gov/pmc/articles/PMC5581518/). 2017 Sep; 108(9): 1811–1819. doi: [10.1111/cas.13315](https://dx.doi.org/10.1111%2Fcas.13315). Downregulation of ULK1 by microRNA‐372 inhibits the survival of human pancreatic adenocarcinoma cells. [Hongxi Chen](https://www.ncbi.nlm.nih.gov/pubmed/?term=Chen%20H%5BAuthor%5D&cauthor=true&cauthor_uid=28677209),  [Zhipeng Zhang](https://www.ncbi.nlm.nih.gov/pubmed/?term=Zhang%20Z%5BAuthor%5D&cauthor=true&cauthor_uid=28677209),  [Yebin Lu](https://www.ncbi.nlm.nih.gov/pubmed/?term=Lu%20Y%5BAuthor%5D&cauthor=true&cauthor_uid=28677209),  [Kun Song](https://www.ncbi.nlm.nih.gov/pubmed/?term=Song%20K%5BAuthor%5D&cauthor=true&cauthor_uid=28677209),  [Xiwu Liu](https://www.ncbi.nlm.nih.gov/pubmed/?term=Liu%20X%5BAuthor%5D&cauthor=true&cauthor_uid=28677209),  [Fada Xia](https://www.ncbi.nlm.nih.gov/pubmed/?term=Xia%20F%5BAuthor%5D&cauthor=true&cauthor_uid=28677209), and [Weijia Sun](https://www.ncbi.nlm.nih.gov/pubmed/?term=Sun%20W%5BAuthor%5D&cauthor=true&cauthor_uid=28677209)
17. [Autophagy.](https://www.ncbi.nlm.nih.gov/pubmed/?term=Ulk1-mediated+phosphorylation+of+AMPK+constitutes+a+negative+regulatory+feedback+loop) 2011 Jul;7(7):696-706. Ulk1-mediated phosphorylation of AMPK constitutes a negative regulatory feedback loop. [Löffler AS](https://www.ncbi.nlm.nih.gov/pubmed/?term=L%C3%B6ffler%20AS%5BAuthor%5D&cauthor=true&cauthor_uid=21460634), [Alers S](https://www.ncbi.nlm.nih.gov/pubmed/?term=Alers%20S%5BAuthor%5D&cauthor=true&cauthor_uid=21460634), [Dieterle AM](https://www.ncbi.nlm.nih.gov/pubmed/?term=Dieterle%20AM%5BAuthor%5D&cauthor=true&cauthor_uid=21460634), [Keppeler H](https://www.ncbi.nlm.nih.gov/pubmed/?term=Keppeler%20H%5BAuthor%5D&cauthor=true&cauthor_uid=21460634), [Franz-Wachtel M](https://www.ncbi.nlm.nih.gov/pubmed/?term=Franz-Wachtel%20M%5BAuthor%5D&cauthor=true&cauthor_uid=21460634), [Kundu M](https://www.ncbi.nlm.nih.gov/pubmed/?term=Kundu%20M%5BAuthor%5D&cauthor=true&cauthor_uid=21460634), [Campbell DG](https://www.ncbi.nlm.nih.gov/pubmed/?term=Campbell%20DG%5BAuthor%5D&cauthor=true&cauthor_uid=21460634), [Wesselborg S](https://www.ncbi.nlm.nih.gov/pubmed/?term=Wesselborg%20S%5BAuthor%5D&cauthor=true&cauthor_uid=21460634), [Alessi DR](https://www.ncbi.nlm.nih.gov/pubmed/?term=Alessi%20DR%5BAuthor%5D&cauthor=true&cauthor_uid=21460634), [Stork B](https://www.ncbi.nlm.nih.gov/pubmed/?term=Stork%20B%5BAuthor%5D&cauthor=true&cauthor_uid=21460634).
18. [Mol Cell. 2015 Jul 16; 59(2): 285–297.](https://www.ncbi.nlm.nih.gov/entrez/eutils/elink.fcgi?dbfrom=pubmed&retmode=ref&cmd=prlinks&id=26118643) doi: [10.1016/j.molcel.2015.05.031](https://dx.doi.org/10.1016%2Fj.molcel.2015.05.031). Small molecule inhibition of the autophagy kinase ULK1 and identification of ULK1 substrates. [Daniel F. Egan](https://www.ncbi.nlm.nih.gov/pubmed/?term=Egan%20DF%5BAuthor%5D&cauthor=true&cauthor_uid=26118643),  [Matthew G.H. Chun](https://www.ncbi.nlm.nih.gov/pubmed/?term=Chun%20MG%5BAuthor%5D&cauthor=true&cauthor_uid=26118643), [Mitchell Vamos](https://www.ncbi.nlm.nih.gov/pubmed/?term=Vamos%20M%5BAuthor%5D&cauthor=true&cauthor_uid=26118643), [Haixia Zou](https://www.ncbi.nlm.nih.gov/pubmed/?term=Zou%20H%5BAuthor%5D&cauthor=true&cauthor_uid=26118643), [Juan Rong](https://www.ncbi.nlm.nih.gov/pubmed/?term=Rong%20J%5BAuthor%5D&cauthor=true&cauthor_uid=26118643), [Chad J. Miller](https://www.ncbi.nlm.nih.gov/pubmed/?term=Miller%20CJ%5BAuthor%5D&cauthor=true&cauthor_uid=26118643), [Hua Jane Lou](https://www.ncbi.nlm.nih.gov/pubmed/?term=Lou%20HJ%5BAuthor%5D&cauthor=true&cauthor_uid=26118643),  [Dhanya Raveendra-Panickar](https://www.ncbi.nlm.nih.gov/pubmed/?term=Raveendra-Panickar%20D%5BAuthor%5D&cauthor=true&cauthor_uid=26118643), [Chih-Cheng Yang](https://www.ncbi.nlm.nih.gov/pubmed/?term=Yang%20CC%5BAuthor%5D&cauthor=true&cauthor_uid=26118643), [Douglas J. Sheffler](https://www.ncbi.nlm.nih.gov/pubmed/?term=Sheffler%20DJ%5BAuthor%5D&cauthor=true&cauthor_uid=26118643), [Peter Teriete](https://www.ncbi.nlm.nih.gov/pubmed/?term=Teriete%20P%5BAuthor%5D&cauthor=true&cauthor_uid=26118643), [John M. Asara](https://www.ncbi.nlm.nih.gov/pubmed/?term=Asara%20JM%5BAuthor%5D&cauthor=true&cauthor_uid=26118643), [Benjamin E. Turk](https://www.ncbi.nlm.nih.gov/pubmed/?term=Turk%20BE%5BAuthor%5D&cauthor=true&cauthor_uid=26118643), [Nicholas D. P. Cosford](https://www.ncbi.nlm.nih.gov/pubmed/?term=Cosford%20ND%5BAuthor%5D&cauthor=true&cauthor_uid=26118643), and [Reuben J. Shaw](https://www.ncbi.nlm.nih.gov/pubmed/?term=Shaw%20RJ%5BAuthor%5D&cauthor=true&cauthor_uid=26118643)
19. [Mol Biol Cell](https://www.ncbi.nlm.nih.gov/pmc/articles/PMC2663920/). 2009 Apr 1; 20(7): 1992–2003. doi: [10.1091/mbc.E08-12-1249](https://dx.doi.org/10.1091%2Fmbc.E08-12-1249). ULK-Atg13-FIP200 Complexes Mediate mTOR Signaling to the Autophagy Machinery. [Chang Hwa Jung](https://www.ncbi.nlm.nih.gov/pubmed/?term=Jung%20CH%5BAuthor%5D&cauthor=true&cauthor_uid=19225151), [Chang Bong Jun](https://www.ncbi.nlm.nih.gov/pubmed/?term=Jun%20CB%5BAuthor%5D&cauthor=true&cauthor_uid=19225151), [Seung-Hyun Ro](https://www.ncbi.nlm.nih.gov/pubmed/?term=Ro%20SH%5BAuthor%5D&cauthor=true&cauthor_uid=19225151), [Young-Mi Kim](https://www.ncbi.nlm.nih.gov/pubmed/?term=Kim%20YM%5BAuthor%5D&cauthor=true&cauthor_uid=19225151), [Neil Michael Otto](https://www.ncbi.nlm.nih.gov/pubmed/?term=Otto%20NM%5BAuthor%5D&cauthor=true&cauthor_uid=19225151), [Jing Cao](https://www.ncbi.nlm.nih.gov/pubmed/?term=Cao%20J%5BAuthor%5D&cauthor=true&cauthor_uid=19225151), [Mondira Kundu](https://www.ncbi.nlm.nih.gov/pubmed/?term=Kundu%20M%5BAuthor%5D&cauthor=true&cauthor_uid=19225151), and [Do-Hyung Kim](https://www.ncbi.nlm.nih.gov/pubmed/?term=Kim%20DH%5BAuthor%5D&cauthor=true&cauthor_uid=19225151)
20. [Nat Commun.](https://www.ncbi.nlm.nih.gov/pubmed/?term=Ulk1-mediated+Atg5-independent+macroautophagy+mediates+elimination+of+mitochondria+from+embryonic+reticulocytes) 2014 Jun 4;5:4004. doi: 10.1038/ncomms5004. Ulk1-mediated Atg5-independent macroautophagy mediates elimination of mitochondria from embryonic reticulocytes. [Honda S](https://www.ncbi.nlm.nih.gov/pubmed/?term=Honda%20S%5BAuthor%5D&cauthor=true&cauthor_uid=24895007), [Arakawa S](https://www.ncbi.nlm.nih.gov/pubmed/?term=Arakawa%20S%5BAuthor%5D&cauthor=true&cauthor_uid=24895007), [Nishida Y](https://www.ncbi.nlm.nih.gov/pubmed/?term=Nishida%20Y%5BAuthor%5D&cauthor=true&cauthor_uid=24895007), [Yamaguchi H](https://www.ncbi.nlm.nih.gov/pubmed/?term=Yamaguchi%20H%5BAuthor%5D&cauthor=true&cauthor_uid=24895007), [Ishii E](https://www.ncbi.nlm.nih.gov/pubmed/?term=Ishii%20E%5BAuthor%5D&cauthor=true&cauthor_uid=24895007), [Shimizu S](https://www.ncbi.nlm.nih.gov/pubmed/?term=Shimizu%20S%5BAuthor%5D&cauthor=true&cauthor_uid=24895007).
21. [Ann Rheum Dis.](https://www.ncbi.nlm.nih.gov/pubmed/24651621) 2015 Jul;74(7):1432-40. doi: 10.1136/annrheumdis-2013-204599. Cartilage-specific deletion of mTOR upregulates autophagy and protects mice from osteoarthritis. [Zhang Y](https://www.ncbi.nlm.nih.gov/pubmed/?term=Zhang%20Y%5BAuthor%5D&cauthor=true&cauthor_uid=24651621), [Vasheghani F](https://www.ncbi.nlm.nih.gov/pubmed/?term=Vasheghani%20F%5BAuthor%5D&cauthor=true&cauthor_uid=24651621), [Li YH](https://www.ncbi.nlm.nih.gov/pubmed/?term=Li%20YH%5BAuthor%5D&cauthor=true&cauthor_uid=24651621), [Blati M](https://www.ncbi.nlm.nih.gov/pubmed/?term=Blati%20M%5BAuthor%5D&cauthor=true&cauthor_uid=24651621), [Simeone K](https://www.ncbi.nlm.nih.gov/pubmed/?term=Simeone%20K%5BAuthor%5D&cauthor=true&cauthor_uid=24651621), [Fahmi H](https://www.ncbi.nlm.nih.gov/pubmed/?term=Fahmi%20H%5BAuthor%5D&cauthor=true&cauthor_uid=24651621), [Lussier B](https://www.ncbi.nlm.nih.gov/pubmed/?term=Lussier%20B%5BAuthor%5D&cauthor=true&cauthor_uid=24651621), [Roughley P](https://www.ncbi.nlm.nih.gov/pubmed/?term=Roughley%20P%5BAuthor%5D&cauthor=true&cauthor_uid=24651621), [Lagares D](https://www.ncbi.nlm.nih.gov/pubmed/?term=Lagares%20D%5BAuthor%5D&cauthor=true&cauthor_uid=24651621), [Pelletier JP](https://www.ncbi.nlm.nih.gov/pubmed/?term=Pelletier%20JP%5BAuthor%5D&cauthor=true&cauthor_uid=24651621), [Martel-Pelletier J](https://www.ncbi.nlm.nih.gov/pubmed/?term=Martel-Pelletier%20J%5BAuthor%5D&cauthor=true&cauthor_uid=24651621), [Kapoor M](https://www.ncbi.nlm.nih.gov/pubmed/?term=Kapoor%20M%5BAuthor%5D&cauthor=true&cauthor_uid=24651621).
22. [Autophagy](https://www.ncbi.nlm.nih.gov/pmc/articles/PMC5103336/). 2016; 12(11): 2085–2097. doi: [10.1080/15548627.2016.1226733](https://dx.doi.org/10.1080%2F15548627.2016.1226733) Suppressed translation and ULK1 degradation as potential mechanisms of autophagy limitation under prolonged starvation [Giulia Allavena](https://www.ncbi.nlm.nih.gov/pubmed/?term=Allavena%20G%5BAuthor%5D&cauthor=true&cauthor_uid=27629431), [Caroline Boyd](https://www.ncbi.nlm.nih.gov/pubmed/?term=Boyd%20C%5BAuthor%5D&cauthor=true&cauthor_uid=27629431), [Kyaw Soe Oo](https://www.ncbi.nlm.nih.gov/pubmed/?term=Oo%20KS%5BAuthor%5D&cauthor=true&cauthor_uid=27629431), [Emilia Maellaro](https://www.ncbi.nlm.nih.gov/pubmed/?term=Maellaro%20E%5BAuthor%5D&cauthor=true&cauthor_uid=27629431), [Boris Zhivotovsky](https://www.ncbi.nlm.nih.gov/pubmed/?term=Zhivotovsky%20B%5BAuthor%5D&cauthor=true&cauthor_uid=27629431), and[Vitaliy O. Kaminskyy](https://www.ncbi.nlm.nih.gov/pubmed/?term=Kaminskyy%20VO%5BAuthor%5D&cauthor=true&cauthor_uid=27629431)
23. [Autophagy](https://www.ncbi.nlm.nih.gov/pmc/articles/PMC3590256/). 2013 Mar 1; 9(3): 361–373. doi: [10.4161/auto.23066](https://dx.doi.org/10.4161%2Fauto.23066). Regulation of nutrient-sensitive autophagy by uncoordinated 51-like kinases 1 and 2. [Fiona McAlpine](https://www.ncbi.nlm.nih.gov/pubmed/?term=McAlpine%20F%5BAuthor%5D&cauthor=true&cauthor_uid=23291478), [Leon E. Williamson](https://www.ncbi.nlm.nih.gov/pubmed/?term=Williamson%20LE%5BAuthor%5D&cauthor=true&cauthor_uid=23291478), [Sharon A. Tooze](https://www.ncbi.nlm.nih.gov/pubmed/?term=Tooze%20SA%5BAuthor%5D&cauthor=true&cauthor_uid=23291478), and [Edmond Y.W. Chan](https://www.ncbi.nlm.nih.gov/pubmed/?term=Chan%20EY%5BAuthor%5D&cauthor=true&cauthor_uid=23291478)
24. [Am J Physiol Endocrinol Metab.](https://www.ncbi.nlm.nih.gov/pubmed/24302004) 2014 Jan 15;306(2):E197-209. doi: 10.1152/ajpendo.00202.2013. Suppression of the mTORC1/STAT3/Notch1 pathway by activated AMPK prevents hepatic insulin resistance induced by excess amino acids. [Li H](https://www.ncbi.nlm.nih.gov/pubmed/?term=Li%20H%5BAuthor%5D&cauthor=true&cauthor_uid=24302004), [Lee J](https://www.ncbi.nlm.nih.gov/pubmed/?term=Lee%20J%5BAuthor%5D&cauthor=true&cauthor_uid=24302004), [He C](https://www.ncbi.nlm.nih.gov/pubmed/?term=He%20C%5BAuthor%5D&cauthor=true&cauthor_uid=24302004), [Zou MH](https://www.ncbi.nlm.nih.gov/pubmed/?term=Zou%20MH%5BAuthor%5D&cauthor=true&cauthor_uid=24302004), [Xie Z](https://www.ncbi.nlm.nih.gov/pubmed/?term=Xie%20Z%5BAuthor%5D&cauthor=true&cauthor_uid=24302004).
25. [Autophagy.](https://www.ncbi.nlm.nih.gov/pubmed/24189100) 2013 Dec;9(12):2069-86. doi: 10.4161/auto.26447. MTOR inhibition attenuates DNA damage and apoptosis through autophagy-mediated suppression of CREB1. [Wang Y](https://www.ncbi.nlm.nih.gov/pubmed/?term=Wang%20Y%5BAuthor%5D&cauthor=true&cauthor_uid=24189100), [Hu Z](https://www.ncbi.nlm.nih.gov/pubmed/?term=Hu%20Z%5BAuthor%5D&cauthor=true&cauthor_uid=24189100), [Liu Z](https://www.ncbi.nlm.nih.gov/pubmed/?term=Liu%20Z%5BAuthor%5D&cauthor=true&cauthor_uid=24189100), [Chen R](https://www.ncbi.nlm.nih.gov/pubmed/?term=Chen%20R%5BAuthor%5D&cauthor=true&cauthor_uid=24189100), [Peng H](https://www.ncbi.nlm.nih.gov/pubmed/?term=Peng%20H%5BAuthor%5D&cauthor=true&cauthor_uid=24189100), [Guo J](https://www.ncbi.nlm.nih.gov/pubmed/?term=Guo%20J%5BAuthor%5D&cauthor=true&cauthor_uid=24189100), [Chen X](https://www.ncbi.nlm.nih.gov/pubmed/?term=Chen%20X%5BAuthor%5D&cauthor=true&cauthor_uid=24189100), [Zhang H](https://www.ncbi.nlm.nih.gov/pubmed/?term=Zhang%20H%5BAuthor%5D&cauthor=true&cauthor_uid=24189100).
26. [J Biol Chem.](https://www.ncbi.nlm.nih.gov/pubmed/26801615) 2016 Mar 11;291(11):6026-35. doi: 10.1074/jbc.M115.689646. Nutrient-regulated Phosphorylation of ATG13 Inhibits Starvation-induced Autophagy. [Puente C](https://www.ncbi.nlm.nih.gov/pubmed/?term=Puente%20C%5BAuthor%5D&cauthor=true&cauthor_uid=26801615), [Hendrickson RC](https://www.ncbi.nlm.nih.gov/pubmed/?term=Hendrickson%20RC%5BAuthor%5D&cauthor=true&cauthor_uid=26801615), [Jiang X](https://www.ncbi.nlm.nih.gov/pubmed/?term=Jiang%20X%5BAuthor%5D&cauthor=true&cauthor_uid=26801615).
27. [EMBO J.](https://www.ncbi.nlm.nih.gov/pubmed/30237309) 2018 Nov 2;37(21). pii: e98589. doi: 10.15252/embj.201798589. Tuberous sclerosis complex is required for tumor maintenance in MYC-driven Burkitt's lymphoma. [Hartleben G](https://www.ncbi.nlm.nih.gov/pubmed/?term=Hartleben%20G%5BAuthor%5D&cauthor=true&cauthor_uid=30237309), [Müller C](https://www.ncbi.nlm.nih.gov/pubmed/?term=M%C3%BCller%20C%5BAuthor%5D&cauthor=true&cauthor_uid=30237309), [Krämer A](https://www.ncbi.nlm.nih.gov/pubmed/?term=Kr%C3%A4mer%20A%5BAuthor%5D&cauthor=true&cauthor_uid=30237309), [Schimmel H](https://www.ncbi.nlm.nih.gov/pubmed/?term=Schimmel%20H%5BAuthor%5D&cauthor=true&cauthor_uid=30237309), [Zidek LM](https://www.ncbi.nlm.nih.gov/pubmed/?term=Zidek%20LM%5BAuthor%5D&cauthor=true&cauthor_uid=30237309), [Dornblut C](https://www.ncbi.nlm.nih.gov/pubmed/?term=Dornblut%20C%5BAuthor%5D&cauthor=true&cauthor_uid=30237309), [Winkler R](https://www.ncbi.nlm.nih.gov/pubmed/?term=Winkler%20R%5BAuthor%5D&cauthor=true&cauthor_uid=30237309), [Eichwald S](https://www.ncbi.nlm.nih.gov/pubmed/?term=Eichwald%20S%5BAuthor%5D&cauthor=true&cauthor_uid=30237309), [Kortman G](https://www.ncbi.nlm.nih.gov/pubmed/?term=Kortman%20G%5BAuthor%5D&cauthor=true&cauthor_uid=30237309), [Kosan C](https://www.ncbi.nlm.nih.gov/pubmed/?term=Kosan%20C%5BAuthor%5D&cauthor=true&cauthor_uid=30237309), [Kluiver J](https://www.ncbi.nlm.nih.gov/pubmed/?term=Kluiver%20J%5BAuthor%5D&cauthor=true&cauthor_uid=30237309), [Petersen I](https://www.ncbi.nlm.nih.gov/pubmed/?term=Petersen%20I%5BAuthor%5D&cauthor=true&cauthor_uid=30237309), [van den Berg A](https://www.ncbi.nlm.nih.gov/pubmed/?term=van%20den%20Berg%20A%5BAuthor%5D&cauthor=true&cauthor_uid=30237309), [Wang ZQ](https://www.ncbi.nlm.nih.gov/pubmed/?term=Wang%20ZQ%5BAuthor%5D&cauthor=true&cauthor_uid=30237309), [Calkhoven CF](https://www.ncbi.nlm.nih.gov/pubmed/?term=Calkhoven%20CF%5BAuthor%5D&cauthor=true&cauthor_uid=30237309).
28. [Int J Mol Sci.](https://www.ncbi.nlm.nih.gov/pubmed/25826530) 2015 Mar 27;16(4):7015-26. doi: 10.3390/ijms16047015. Identification of an AMPK phosphorylation site in Drosophila TSC2 (gigas) that regulate cell growth. [Kim M](https://www.ncbi.nlm.nih.gov/pubmed/?term=Kim%20M%5BAuthor%5D&cauthor=true&cauthor_uid=25826530), [Lee JH](https://www.ncbi.nlm.nih.gov/pubmed/?term=Lee%20JH%5BAuthor%5D&cauthor=true&cauthor_uid=25826530).
29. [Eur J Cancer.](https://www.ncbi.nlm.nih.gov/pubmed/20656472) 2010 Oct;46(15):2806-20. doi: 10.1016/j.ejca.2010.06.117. Novel mechanism of reducing tumourigenesis: upregulation of the DNA repair enzyme OGG1 by rapamycin-mediated AMPK activation and mTOR inhibition. [Habib SL](https://www.ncbi.nlm.nih.gov/pubmed/?term=Habib%20SL%5BAuthor%5D&cauthor=true&cauthor_uid=20656472), [Kasinath BS](https://www.ncbi.nlm.nih.gov/pubmed/?term=Kasinath%20BS%5BAuthor%5D&cauthor=true&cauthor_uid=20656472), [Arya RR](https://www.ncbi.nlm.nih.gov/pubmed/?term=Arya%20RR%5BAuthor%5D&cauthor=true&cauthor_uid=20656472), [Vexler S](https://www.ncbi.nlm.nih.gov/pubmed/?term=Vexler%20S%5BAuthor%5D&cauthor=true&cauthor_uid=20656472), [Velagapudi C](https://www.ncbi.nlm.nih.gov/pubmed/?term=Velagapudi%20C%5BAuthor%5D&cauthor=true&cauthor_uid=20656472).
30. Cell Cycle[Cell Cycle.](https://www.ncbi.nlm.nih.gov/pubmed/24304514) 2014;13(3):371-82. doi: 10.4161/cc.27355. Resveratrol prevents rapamycin-induced upregulation of autophagy and selectively induces apoptosis in TSC2-deficient cells. [Alayev A](https://www.ncbi.nlm.nih.gov/pubmed/?term=Alayev%20A%5BAuthor%5D&cauthor=true&cauthor_uid=24304514), [Sun Y](https://www.ncbi.nlm.nih.gov/pubmed/?term=Sun%20Y%5BAuthor%5D&cauthor=true&cauthor_uid=24304514), [Snyder RB](https://www.ncbi.nlm.nih.gov/pubmed/?term=Snyder%20RB%5BAuthor%5D&cauthor=true&cauthor_uid=24304514), [Berger SM](https://www.ncbi.nlm.nih.gov/pubmed/?term=Berger%20SM%5BAuthor%5D&cauthor=true&cauthor_uid=24304514), [Yu JJ](https://www.ncbi.nlm.nih.gov/pubmed/?term=Yu%20JJ%5BAuthor%5D&cauthor=true&cauthor_uid=24304514), [Holz MK](https://www.ncbi.nlm.nih.gov/pubmed/?term=Holz%20MK%5BAuthor%5D&cauthor=true&cauthor_uid=24304514).
31. [Biochem Biophys Res Commun.](https://www.ncbi.nlm.nih.gov/pubmed/28082200) 2017 Feb 5;483(2):897-903. doi: 10.1016/j.bbrc.2017.01.031. mTOR up-regulation of PFKFB3 is essential for acute myeloid leukemia cell survival. [Feng Y](https://www.ncbi.nlm.nih.gov/pubmed/?term=Feng%20Y%5BAuthor%5D&cauthor=true&cauthor_uid=28082200), [Wu L](https://www.ncbi.nlm.nih.gov/pubmed/?term=Wu%20L%5BAuthor%5D&cauthor=true&cauthor_uid=28082200).
32. [J Neurosci.](https://www.ncbi.nlm.nih.gov/pubmed/23136410) 2012 Nov 7;32(45):15704-14. doi: 10.1523/JNEUROSCI.2392-12.2012. Impaired autophagy in neurons after disinhibition of mammalian target of rapamycin and its contribution to epileptogenesis. [McMahon J](https://www.ncbi.nlm.nih.gov/pubmed/?term=McMahon%20J%5BAuthor%5D&cauthor=true&cauthor_uid=23136410), [Huang X](https://www.ncbi.nlm.nih.gov/pubmed/?term=Huang%20X%5BAuthor%5D&cauthor=true&cauthor_uid=23136410), [Yang J](https://www.ncbi.nlm.nih.gov/pubmed/?term=Yang%20J%5BAuthor%5D&cauthor=true&cauthor_uid=23136410), [Komatsu M](https://www.ncbi.nlm.nih.gov/pubmed/?term=Komatsu%20M%5BAuthor%5D&cauthor=true&cauthor_uid=23136410), [Yue Z](https://www.ncbi.nlm.nih.gov/pubmed/?term=Yue%20Z%5BAuthor%5D&cauthor=true&cauthor_uid=23136410), [Qian J](https://www.ncbi.nlm.nih.gov/pubmed/?term=Qian%20J%5BAuthor%5D&cauthor=true&cauthor_uid=23136410), [Zhu X](https://www.ncbi.nlm.nih.gov/pubmed/?term=Zhu%20X%5BAuthor%5D&cauthor=true&cauthor_uid=23136410), [Huang Y](https://www.ncbi.nlm.nih.gov/pubmed/?term=Huang%20Y%5BAuthor%5D&cauthor=true&cauthor_uid=23136410).
33. [BMC Biochem.](https://www.ncbi.nlm.nih.gov/pubmed/27387347) 2016 Jul 7;17(1):14. doi: 10.1186/s12858-016-0069-6. Sustained activation of mTORC1 in macrophages increases AMPKα-dependent autophagy to maintain cellular homeostasis. [Pan H](https://www.ncbi.nlm.nih.gov/pubmed/?term=Pan%20H%5BAuthor%5D&cauthor=true&cauthor_uid=27387347), [Zhong XP](https://www.ncbi.nlm.nih.gov/pubmed/?term=Zhong%20XP%5BAuthor%5D&cauthor=true&cauthor_uid=27387347), [Lee S](https://www.ncbi.nlm.nih.gov/pubmed/?term=Lee%20S%5BAuthor%5D&cauthor=true&cauthor_uid=27387347).
34. [Cell Metab.](https://www.ncbi.nlm.nih.gov/pubmed/23602450) 2013 May 7;17(5):731-44. doi: 10.1016/j.cmet.2013.03.015. Sustained activation of mTORC1 in skeletal muscle inhibits constitutive and starvation-induced autophagy and causes a severe, late-onset myopathy. [Castets P](https://www.ncbi.nlm.nih.gov/pubmed/?term=Castets%20P%5BAuthor%5D&cauthor=true&cauthor_uid=23602450), [Lin S](https://www.ncbi.nlm.nih.gov/pubmed/?term=Lin%20S%5BAuthor%5D&cauthor=true&cauthor_uid=23602450), [Rion N](https://www.ncbi.nlm.nih.gov/pubmed/?term=Rion%20N%5BAuthor%5D&cauthor=true&cauthor_uid=23602450), [Di Fulvio S](https://www.ncbi.nlm.nih.gov/pubmed/?term=Di%20Fulvio%20S%5BAuthor%5D&cauthor=true&cauthor_uid=23602450), [Romanino K](https://www.ncbi.nlm.nih.gov/pubmed/?term=Romanino%20K%5BAuthor%5D&cauthor=true&cauthor_uid=23602450), [Guridi M](https://www.ncbi.nlm.nih.gov/pubmed/?term=Guridi%20M%5BAuthor%5D&cauthor=true&cauthor_uid=23602450), [Frank S](https://www.ncbi.nlm.nih.gov/pubmed/?term=Frank%20S%5BAuthor%5D&cauthor=true&cauthor_uid=23602450), [Tintignac LA](https://www.ncbi.nlm.nih.gov/pubmed/?term=Tintignac%20LA%5BAuthor%5D&cauthor=true&cauthor_uid=23602450), [Sinnreich M](https://www.ncbi.nlm.nih.gov/pubmed/?term=Sinnreich%20M%5BAuthor%5D&cauthor=true&cauthor_uid=23602450), [Rüegg MA](https://www.ncbi.nlm.nih.gov/pubmed/?term=R%C3%BCegg%20MA%5BAuthor%5D&cauthor=true&cauthor_uid=23602450).
35. [Biochim Biophys Acta.](https://www.ncbi.nlm.nih.gov/pubmed/27542907) 2016 Nov;1863(11):2658-2667. doi: 10.1016/j.bbamcr.2016.08.006. TSC2 N-terminal lysine acetylation status affects to its stability modulating mTORC1 signaling and autophagy. [García-Aguilar A](https://www.ncbi.nlm.nih.gov/pubmed/?term=Garc%C3%ADa-Aguilar%20A%5BAuthor%5D&cauthor=true&cauthor_uid=27542907), [Guillén C](https://www.ncbi.nlm.nih.gov/pubmed/?term=Guill%C3%A9n%20C%5BAuthor%5D&cauthor=true&cauthor_uid=27542907), [Nellist M](https://www.ncbi.nlm.nih.gov/pubmed/?term=Nellist%20M%5BAuthor%5D&cauthor=true&cauthor_uid=27542907), [Bartolomé A](https://www.ncbi.nlm.nih.gov/pubmed/?term=Bartolom%C3%A9%20A%5BAuthor%5D&cauthor=true&cauthor_uid=27542907), [Benito M](https://www.ncbi.nlm.nih.gov/pubmed/?term=Benito%20M%5BAuthor%5D&cauthor=true&cauthor_uid=27542907).
36. [J Cell Physiol.](https://www.ncbi.nlm.nih.gov/pubmed/27225870) 2017 Feb;232(2):436-446. doi: 10.1002/jcp.25443. Combination of Rapamycin and Resveratrol for Treatment of Bladder Cancer. [Alayev A](https://www.ncbi.nlm.nih.gov/pubmed/?term=Alayev%20A%5BAuthor%5D&cauthor=true&cauthor_uid=27225870), [Salamon RS](https://www.ncbi.nlm.nih.gov/pubmed/?term=Salamon%20RS%5BAuthor%5D&cauthor=true&cauthor_uid=27225870), [Schwartz NS](https://www.ncbi.nlm.nih.gov/pubmed/?term=Schwartz%20NS%5BAuthor%5D&cauthor=true&cauthor_uid=27225870), [Berman AY](https://www.ncbi.nlm.nih.gov/pubmed/?term=Berman%20AY%5BAuthor%5D&cauthor=true&cauthor_uid=27225870), [Wiener SL](https://www.ncbi.nlm.nih.gov/pubmed/?term=Wiener%20SL%5BAuthor%5D&cauthor=true&cauthor_uid=27225870), [Holz MK](https://www.ncbi.nlm.nih.gov/pubmed/?term=Holz%20MK%5BAuthor%5D&cauthor=true&cauthor_uid=27225870).
37. [J Biol Chem.](https://www.ncbi.nlm.nih.gov/pubmed/20851890) 2010 Nov 19;285(47):36387-94. doi: 10.1074/jbc.M110.169284. Resveratrol inhibits mTOR signaling by promoting the interaction between mTOR and DEPTOR. [Liu M](https://www.ncbi.nlm.nih.gov/pubmed/?term=Liu%20M%5BAuthor%5D&cauthor=true&cauthor_uid=20851890), [Wilk SA](https://www.ncbi.nlm.nih.gov/pubmed/?term=Wilk%20SA%5BAuthor%5D&cauthor=true&cauthor_uid=20851890), [Wang A](https://www.ncbi.nlm.nih.gov/pubmed/?term=Wang%20A%5BAuthor%5D&cauthor=true&cauthor_uid=20851890), [Zhou L](https://www.ncbi.nlm.nih.gov/pubmed/?term=Zhou%20L%5BAuthor%5D&cauthor=true&cauthor_uid=20851890), [Wang RH](https://www.ncbi.nlm.nih.gov/pubmed/?term=Wang%20RH%5BAuthor%5D&cauthor=true&cauthor_uid=20851890), [Ogawa W](https://www.ncbi.nlm.nih.gov/pubmed/?term=Ogawa%20W%5BAuthor%5D&cauthor=true&cauthor_uid=20851890), [Deng C](https://www.ncbi.nlm.nih.gov/pubmed/?term=Deng%20C%5BAuthor%5D&cauthor=true&cauthor_uid=20851890), [Dong LQ](https://www.ncbi.nlm.nih.gov/pubmed/?term=Dong%20LQ%5BAuthor%5D&cauthor=true&cauthor_uid=20851890), [Liu F](https://www.ncbi.nlm.nih.gov/pubmed/?term=Liu%20F%5BAuthor%5D&cauthor=true&cauthor_uid=20851890).
38. [J Biol Chem](https://www.ncbi.nlm.nih.gov/pmc/articles/PMC3887183/). 2014 Jan 10; 289(2): 1164–1173. doi: [10.1074/jbc.M113.526335](https://dx.doi.org/10.1074%2Fjbc.M113.526335). Hyperactivation of Mammalian Target of Rapamycin Complex 1 (mTORC1) Promotes Breast Cancer Progression through Enhancing Glucose Starvation-induced Autophagy and Akt Signaling. [Yongqiang Chen](https://www.ncbi.nlm.nih.gov/pubmed/?term=Chen%20Y%5BAuthor%5D&cauthor=true&cauthor_uid=24275666), [Huijun Wei](https://www.ncbi.nlm.nih.gov/pubmed/?term=Wei%20H%5BAuthor%5D&cauthor=true&cauthor_uid=24275666), [Fei Liu](https://www.ncbi.nlm.nih.gov/pubmed/?term=Liu%20F%5BAuthor%5D&cauthor=true&cauthor_uid=24275666), and [Jun-Lin Guan](https://www.ncbi.nlm.nih.gov/pubmed/?term=Guan%20JL%5BAuthor%5D&cauthor=true&cauthor_uid=24275666)
39. [Brain.](https://www.ncbi.nlm.nih.gov/pubmed/28969371) 2017 Oct 1;140(10):2623-2638. doi: 10.1093/brain/awx196. Mammalian target of rapamycin complex 1 activation sensitizes human glioma cells to hypoxia-induced cell death. [Thiepold AL](https://www.ncbi.nlm.nih.gov/pubmed/?term=Thiepold%20AL%5BAuthor%5D&cauthor=true&cauthor_uid=28969371), [Lorenz NI](https://www.ncbi.nlm.nih.gov/pubmed/?term=Lorenz%20NI%5BAuthor%5D&cauthor=true&cauthor_uid=28969371), [Foltyn M](https://www.ncbi.nlm.nih.gov/pubmed/?term=Foltyn%20M%5BAuthor%5D&cauthor=true&cauthor_uid=28969371), [Engel AL](https://www.ncbi.nlm.nih.gov/pubmed/?term=Engel%20AL%5BAuthor%5D&cauthor=true&cauthor_uid=28969371), [Divé I](https://www.ncbi.nlm.nih.gov/pubmed/?term=Div%C3%A9%20I%5BAuthor%5D&cauthor=true&cauthor_uid=28969371), [Urban H](https://www.ncbi.nlm.nih.gov/pubmed/?term=Urban%20H%5BAuthor%5D&cauthor=true&cauthor_uid=28969371), [Heller S](https://www.ncbi.nlm.nih.gov/pubmed/?term=Heller%20S%5BAuthor%5D&cauthor=true&cauthor_uid=28969371), [Bruns I](https://www.ncbi.nlm.nih.gov/pubmed/?term=Bruns%20I%5BAuthor%5D&cauthor=true&cauthor_uid=28969371), [Hofmann U](https://www.ncbi.nlm.nih.gov/pubmed/?term=Hofmann%20U%5BAuthor%5D&cauthor=true&cauthor_uid=28969371), [Dröse S](https://www.ncbi.nlm.nih.gov/pubmed/?term=Dr%C3%B6se%20S%5BAuthor%5D&cauthor=true&cauthor_uid=28969371), [Harter PN](https://www.ncbi.nlm.nih.gov/pubmed/?term=Harter%20PN%5BAuthor%5D&cauthor=true&cauthor_uid=28969371), [Mittelbronn M](https://www.ncbi.nlm.nih.gov/pubmed/?term=Mittelbronn%20M%5BAuthor%5D&cauthor=true&cauthor_uid=28969371), [Steinbach JP](https://www.ncbi.nlm.nih.gov/pubmed/?term=Steinbach%20JP%5BAuthor%5D&cauthor=true&cauthor_uid=28969371), [Ronellenfitsch MW](https://www.ncbi.nlm.nih.gov/pubmed/?term=Ronellenfitsch%20MW%5BAuthor%5D&cauthor=true&cauthor_uid=28969371).
40. [Cell.](https://www.ncbi.nlm.nih.gov/pubmed/24529380) 2014 Feb 13;156(4):786-99. doi: 10.1016/j.cell.2014.01.024. Regulation of TORC1 in response to amino acid starvation via lysosomal recruitment of TSC2. [Demetriades C](https://www.ncbi.nlm.nih.gov/pubmed/?term=Demetriades%20C%5BAuthor%5D&cauthor=true&cauthor_uid=24529380), [Doumpas N](https://www.ncbi.nlm.nih.gov/pubmed/?term=Doumpas%20N%5BAuthor%5D&cauthor=true&cauthor_uid=24529380), [Teleman AA](https://www.ncbi.nlm.nih.gov/pubmed/?term=Teleman%20AA%5BAuthor%5D&cauthor=true&cauthor_uid=24529380).
41. [J Biol Chem.](https://www.ncbi.nlm.nih.gov/pubmed/24275666) 2014 Jan 10;289(2):1164-73. doi: 10.1074/jbc.M113.526335. Hyperactivation of mammalian target of rapamycin complex 1 (mTORC1) promotes breast cancer progression through enhancing glucose starvation-induced autophagy and Akt signaling. [Chen Y](https://www.ncbi.nlm.nih.gov/pubmed/?term=Chen%20Y%5BAuthor%5D&cauthor=true&cauthor_uid=24275666), [Wei H](https://www.ncbi.nlm.nih.gov/pubmed/?term=Wei%20H%5BAuthor%5D&cauthor=true&cauthor_uid=24275666), [Liu F](https://www.ncbi.nlm.nih.gov/pubmed/?term=Liu%20F%5BAuthor%5D&cauthor=true&cauthor_uid=24275666), [Guan JL](https://www.ncbi.nlm.nih.gov/pubmed/?term=Guan%20JL%5BAuthor%5D&cauthor=true&cauthor_uid=24275666).
42. [Autophagy.](https://www.ncbi.nlm.nih.gov/pubmed/21808151) 2011 Oct;7(10):1173-86. doi: 10.4161/auto.7.10.16681. Impaired autophagy due to constitutive mTOR activation sensitizes TSC2-null cells to cell death under stress. [Ng S](https://www.ncbi.nlm.nih.gov/pubmed/?term=Ng%20S%5BAuthor%5D&cauthor=true&cauthor_uid=21808151), [Wu YT](https://www.ncbi.nlm.nih.gov/pubmed/?term=Wu%20YT%5BAuthor%5D&cauthor=true&cauthor_uid=21808151), [Chen B](https://www.ncbi.nlm.nih.gov/pubmed/?term=Chen%20B%5BAuthor%5D&cauthor=true&cauthor_uid=21808151), [Zhou J](https://www.ncbi.nlm.nih.gov/pubmed/?term=Zhou%20J%5BAuthor%5D&cauthor=true&cauthor_uid=21808151), [Shen HM](https://www.ncbi.nlm.nih.gov/pubmed/?term=Shen%20HM%5BAuthor%5D&cauthor=true&cauthor_uid=21808151).
43. [EMBO J.](https://www.ncbi.nlm.nih.gov/pubmed/17962806) 2007 Nov 28;26(23):4812-23. Constitutive mTOR activation in TSC mutants sensitizes cells to energy starvation and genomic damage via p53. [Lee CH](https://www.ncbi.nlm.nih.gov/pubmed/?term=Lee%20CH%5BAuthor%5D&cauthor=true&cauthor_uid=17962806), [Inoki K](https://www.ncbi.nlm.nih.gov/pubmed/?term=Inoki%20K%5BAuthor%5D&cauthor=true&cauthor_uid=17962806), [Karbowniczek M](https://www.ncbi.nlm.nih.gov/pubmed/?term=Karbowniczek%20M%5BAuthor%5D&cauthor=true&cauthor_uid=17962806), [Petroulakis E](https://www.ncbi.nlm.nih.gov/pubmed/?term=Petroulakis%20E%5BAuthor%5D&cauthor=true&cauthor_uid=17962806), [Sonenberg N](https://www.ncbi.nlm.nih.gov/pubmed/?term=Sonenberg%20N%5BAuthor%5D&cauthor=true&cauthor_uid=17962806), [Henske EP](https://www.ncbi.nlm.nih.gov/pubmed/?term=Henske%20EP%5BAuthor%5D&cauthor=true&cauthor_uid=17962806), [Guan KL](https://www.ncbi.nlm.nih.gov/pubmed/?term=Guan%20KL%5BAuthor%5D&cauthor=true&cauthor_uid=17962806).
44. [Nat Cell Biol.](https://www.ncbi.nlm.nih.gov/pubmed/12172555) 2002 Sep;4(9):699-704. Tsc tumour suppressor proteins antagonize amino-acid-TOR signalling. [Gao X](https://www.ncbi.nlm.nih.gov/pubmed/?term=Gao%20X%5BAuthor%5D&cauthor=true&cauthor_uid=12172555), [Zhang Y](https://www.ncbi.nlm.nih.gov/pubmed/?term=Zhang%20Y%5BAuthor%5D&cauthor=true&cauthor_uid=12172555), [Arrazola P](https://www.ncbi.nlm.nih.gov/pubmed/?term=Arrazola%20P%5BAuthor%5D&cauthor=true&cauthor_uid=12172555), [Hino O](https://www.ncbi.nlm.nih.gov/pubmed/?term=Hino%20O%5BAuthor%5D&cauthor=true&cauthor_uid=12172555), [Kobayashi T](https://www.ncbi.nlm.nih.gov/pubmed/?term=Kobayashi%20T%5BAuthor%5D&cauthor=true&cauthor_uid=12172555), [Yeung RS](https://www.ncbi.nlm.nih.gov/pubmed/?term=Yeung%20RS%5BAuthor%5D&cauthor=true&cauthor_uid=12172555), [Ru B](https://www.ncbi.nlm.nih.gov/pubmed/?term=Ru%20B%5BAuthor%5D&cauthor=true&cauthor_uid=12172555), [Pan D](https://www.ncbi.nlm.nih.gov/pubmed/?term=Pan%20D%5BAuthor%5D&cauthor=true&cauthor_uid=12172555).
45. [Biomed Pharmacother.](https://www.ncbi.nlm.nih.gov/pubmed/?term=Lipopolysaccharide+induces+autophagy+by+targeting+the+AMPK-mTOR+pathway+in+Human+Nasal+Epithelial+Cells) 2017 Dec;96:899-904. doi: 10.1016/j.biopha.2017.12.011. Lipopolysaccharide induces autophagy by targeting the AMPKmTOR pathway in Human NasalEpithelial Cells. [Wang XH](https://www.ncbi.nlm.nih.gov/pubmed/?term=Wang%20XH%5BAuthor%5D&cauthor=true&cauthor_uid=29223553), [Zhang ZH](https://www.ncbi.nlm.nih.gov/pubmed/?term=Zhang%20ZH%5BAuthor%5D&cauthor=true&cauthor_uid=29223553), [Cai XL](https://www.ncbi.nlm.nih.gov/pubmed/?term=Cai%20XL%5BAuthor%5D&cauthor=true&cauthor_uid=29223553), [Ye P](https://www.ncbi.nlm.nih.gov/pubmed/?term=Ye%20P%5BAuthor%5D&cauthor=true&cauthor_uid=29223553), [Feng X](https://www.ncbi.nlm.nih.gov/pubmed/?term=Feng%20X%5BAuthor%5D&cauthor=true&cauthor_uid=29223553), [Liu TT](https://www.ncbi.nlm.nih.gov/pubmed/?term=Liu%20TT%5BAuthor%5D&cauthor=true&cauthor_uid=29223553), [Li XZ](https://www.ncbi.nlm.nih.gov/pubmed/?term=Li%20XZ%5BAuthor%5D&cauthor=true&cauthor_uid=29223553).
46. [Front Med.](https://www.ncbi.nlm.nih.gov/pubmed/?term=Resveratrol+reduces+intracellular+reactive+oxygen+species+levels+by+inducing+autophagy+through+the+AMPK-mTOR+pathway) 2018 Dec;12(6):697-706. doi: 10.1007/s11684-018-0655-7. Resveratrol reduces intracellular reactive oxygen species levels by inducing autophagy through the AMPK-mTOR pathway. [Song J](https://www.ncbi.nlm.nih.gov/pubmed/?term=Song%20J%5BAuthor%5D&cauthor=true&cauthor_uid=30421395), [Huang Y](https://www.ncbi.nlm.nih.gov/pubmed/?term=Huang%20Y%5BAuthor%5D&cauthor=true&cauthor_uid=30421395), [Zheng W](https://www.ncbi.nlm.nih.gov/pubmed/?term=Zheng%20W%5BAuthor%5D&cauthor=true&cauthor_uid=30421395), [Yan J](https://www.ncbi.nlm.nih.gov/pubmed/?term=Yan%20J%5BAuthor%5D&cauthor=true&cauthor_uid=30421395), [Cheng M](https://www.ncbi.nlm.nih.gov/pubmed/?term=Cheng%20M%5BAuthor%5D&cauthor=true&cauthor_uid=30421395), [Zhao R](https://www.ncbi.nlm.nih.gov/pubmed/?term=Zhao%20R%5BAuthor%5D&cauthor=true&cauthor_uid=30421395), [Chen L](https://www.ncbi.nlm.nih.gov/pubmed/?term=Chen%20L%5BAuthor%5D&cauthor=true&cauthor_uid=30421395), [Hu C](https://www.ncbi.nlm.nih.gov/pubmed/?term=Hu%20C%5BAuthor%5D&cauthor=true&cauthor_uid=30421395), [Jia W](https://www.ncbi.nlm.nih.gov/pubmed/?term=Jia%20W%5BAuthor%5D&cauthor=true&cauthor_uid=30421395).
47. [PLoS One.](https://www.ncbi.nlm.nih.gov/pubmed/?term=Compound+C+inhibits+nonsense-mediated+RNA+decay+independently+of+AMPK) 2018 Oct 5;13(10):e0204978. doi: 10.1371/journal.pone.0204978. eCollection 2018. Compound C inhibits nonsense-mediated RNA decay independently of AMPK. [Cheruiyot A](https://www.ncbi.nlm.nih.gov/pubmed/?term=Cheruiyot%20A%5BAuthor%5D&cauthor=true&cauthor_uid=30289931), [Li S](https://www.ncbi.nlm.nih.gov/pubmed/?term=Li%20S%5BAuthor%5D&cauthor=true&cauthor_uid=30289931), [Nickless A](https://www.ncbi.nlm.nih.gov/pubmed/?term=Nickless%20A%5BAuthor%5D&cauthor=true&cauthor_uid=30289931), [Roth R](https://www.ncbi.nlm.nih.gov/pubmed/?term=Roth%20R%5BAuthor%5D&cauthor=true&cauthor_uid=30289931), [Fitzpatrick JAJ](https://www.ncbi.nlm.nih.gov/pubmed/?term=Fitzpatrick%20JAJ%5BAuthor%5D&cauthor=true&cauthor_uid=30289931), [You Z](https://www.ncbi.nlm.nih.gov/pubmed/?term=You%20Z%5BAuthor%5D&cauthor=true&cauthor_uid=30289931).
48. [Oncotarget.](https://www.ncbi.nlm.nih.gov/pubmed/28978086) 2017 Jul 4;8(40):67942-67954. doi: 10.18632/oncotarget.18980. AMPK activation-dependent autophagy compromises oleanolic acid-induced cytotoxicity in human bladder cancer cells. [Song Y](https://www.ncbi.nlm.nih.gov/pubmed/?term=Song%20Y%5BAuthor%5D&cauthor=true&cauthor_uid=28978086), [Zhang P](https://www.ncbi.nlm.nih.gov/pubmed/?term=Zhang%20P%5BAuthor%5D&cauthor=true&cauthor_uid=28978086), [Sun Y](https://www.ncbi.nlm.nih.gov/pubmed/?term=Sun%20Y%5BAuthor%5D&cauthor=true&cauthor_uid=28978086), [Li X](https://www.ncbi.nlm.nih.gov/pubmed/?term=Li%20X%5BAuthor%5D&cauthor=true&cauthor_uid=28978086), [Chen L](https://www.ncbi.nlm.nih.gov/pubmed/?term=Chen%20L%5BAuthor%5D&cauthor=true&cauthor_uid=28978086), [Xiao Y](https://www.ncbi.nlm.nih.gov/pubmed/?term=Xiao%20Y%5BAuthor%5D&cauthor=true&cauthor_uid=28978086), [Xing Y](https://www.ncbi.nlm.nih.gov/pubmed/?term=Xing%20Y%5BAuthor%5D&cauthor=true&cauthor_uid=28978086).
49. [Clin Cancer Res.](https://www.ncbi.nlm.nih.gov/pubmed/27864418) 2017 Jun 1;23(11):2781-2794. doi: 10.1158/1078-0432.CCR-16-1903. AMPK-ULK1-Mediated Autophagy Confers Resistance to BET Inhibitor JQ1 in Acute Myeloid Leukemia Stem Cells. [Jang JE](https://www.ncbi.nlm.nih.gov/pubmed/?term=Jang%20JE%5BAuthor%5D&cauthor=true&cauthor_uid=27864418), [Eom JI](https://www.ncbi.nlm.nih.gov/pubmed/?term=Eom%20JI%5BAuthor%5D&cauthor=true&cauthor_uid=27864418), [Jeung HK](https://www.ncbi.nlm.nih.gov/pubmed/?term=Jeung%20HK%5BAuthor%5D&cauthor=true&cauthor_uid=27864418), [Cheong JW](https://www.ncbi.nlm.nih.gov/pubmed/?term=Cheong%20JW%5BAuthor%5D&cauthor=true&cauthor_uid=27864418), [Lee JY](https://www.ncbi.nlm.nih.gov/pubmed/?term=Lee%20JY%5BAuthor%5D&cauthor=true&cauthor_uid=27864418), [Kim JS](https://www.ncbi.nlm.nih.gov/pubmed/?term=Kim%20JS%5BAuthor%5D&cauthor=true&cauthor_uid=27864418), [Min YH](https://www.ncbi.nlm.nih.gov/pubmed/?term=Min%20YH%5BAuthor%5D&cauthor=true&cauthor_uid=27864418).
50. [J Cell Biochem.](https://www.ncbi.nlm.nih.gov/pubmed/29384220) 2018 Jul;119(7):5538-5550. doi: 10.1002/jcb.26723. Compound C induces protective autophagy in human cholangiocarcinoma cells via Akt/mTOR-independent pathway. [Zhao X](https://www.ncbi.nlm.nih.gov/pubmed/?term=Zhao%20X%5BAuthor%5D&cauthor=true&cauthor_uid=29384220), [Luo G](https://www.ncbi.nlm.nih.gov/pubmed/?term=Luo%20G%5BAuthor%5D&cauthor=true&cauthor_uid=29384220), [Cheng Y](https://www.ncbi.nlm.nih.gov/pubmed/?term=Cheng%20Y%5BAuthor%5D&cauthor=true&cauthor_uid=29384220), [Yu W](https://www.ncbi.nlm.nih.gov/pubmed/?term=Yu%20W%5BAuthor%5D&cauthor=true&cauthor_uid=29384220), [Chen R](https://www.ncbi.nlm.nih.gov/pubmed/?term=Chen%20R%5BAuthor%5D&cauthor=true&cauthor_uid=29384220), [Xiao B](https://www.ncbi.nlm.nih.gov/pubmed/?term=Xiao%20B%5BAuthor%5D&cauthor=true&cauthor_uid=29384220), [Xiang Y](https://www.ncbi.nlm.nih.gov/pubmed/?term=Xiang%20Y%5BAuthor%5D&cauthor=true&cauthor_uid=29384220), [Feng C](https://www.ncbi.nlm.nih.gov/pubmed/?term=Feng%20C%5BAuthor%5D&cauthor=true&cauthor_uid=29384220), [Fu W](https://www.ncbi.nlm.nih.gov/pubmed/?term=Fu%20W%5BAuthor%5D&cauthor=true&cauthor_uid=29384220), [Duan C](https://www.ncbi.nlm.nih.gov/pubmed/?term=Duan%20C%5BAuthor%5D&cauthor=true&cauthor_uid=29384220), [Yao F](https://www.ncbi.nlm.nih.gov/pubmed/?term=Yao%20F%5BAuthor%5D&cauthor=true&cauthor_uid=29384220), [Xia X](https://www.ncbi.nlm.nih.gov/pubmed/?term=Xia%20X%5BAuthor%5D&cauthor=true&cauthor_uid=29384220), [Tao Q](https://www.ncbi.nlm.nih.gov/pubmed/?term=Tao%20Q%5BAuthor%5D&cauthor=true&cauthor_uid=29384220), [Wei M](https://www.ncbi.nlm.nih.gov/pubmed/?term=Wei%20M%5BAuthor%5D&cauthor=true&cauthor_uid=29384220), [Dai R](https://www.ncbi.nlm.nih.gov/pubmed/?term=Dai%20R%5BAuthor%5D&cauthor=true&cauthor_uid=29384220).
51. [Cell Mol Neurobiol.](https://www.ncbi.nlm.nih.gov/pubmed/?term=Shear+Stress+Induces+Phenotypic+Modulation+of+Vascular+Smooth+Muscle+Cells+via+AMPK%2FmTOR%2FULK1-Mediated+Autophagy) 2018 Mar;38(2):541-548. doi: 10.1007/s10571-017-0505-1. Shear Stress Induces Phenotypic Modulation of Vascular Smooth Muscle Cells viaAMPK/mTOR/ULK1-Mediated Autophagy.,[Sun L](https://www.ncbi.nlm.nih.gov/pubmed/?term=Sun%20L%5BAuthor%5D&cauthor=true&cauthor_uid=28560556), [Zhao M](https://www.ncbi.nlm.nih.gov/pubmed/?term=Zhao%20M%5BAuthor%5D&cauthor=true&cauthor_uid=28560556), [Liu A](https://www.ncbi.nlm.nih.gov/pubmed/?term=Liu%20A%5BAuthor%5D&cauthor=true&cauthor_uid=28560556), [Lv M](https://www.ncbi.nlm.nih.gov/pubmed/?term=Lv%20M%5BAuthor%5D&cauthor=true&cauthor_uid=28560556), [Zhang J](https://www.ncbi.nlm.nih.gov/pubmed/?term=Zhang%20J%5BAuthor%5D&cauthor=true&cauthor_uid=28560556), [Li Y](https://www.ncbi.nlm.nih.gov/pubmed/?term=Li%20Y%5BAuthor%5D&cauthor=true&cauthor_uid=28560556), [Yang X](https://www.ncbi.nlm.nih.gov/pubmed/?term=Yang%20X%5BAuthor%5D&cauthor=true&cauthor_uid=28560556), [Wu Z](https://www.ncbi.nlm.nih.gov/pubmed/?term=Wu%20Z%5BAuthor%5D&cauthor=true&cauthor_uid=28560556).
52. [PLoS One.](https://www.ncbi.nlm.nih.gov/pubmed/22514710) 2012;7(4):e35092. doi: 10.1371/journal.pone.0035092. Acute activation of AMP-activated protein kinase prevents H2O2-induced premature senescence in primary human keratinocytes. [Ido Y](https://www.ncbi.nlm.nih.gov/pubmed/?term=Ido%20Y%5BAuthor%5D&cauthor=true&cauthor_uid=22514710), [Duranton A](https://www.ncbi.nlm.nih.gov/pubmed/?term=Duranton%20A%5BAuthor%5D&cauthor=true&cauthor_uid=22514710), [Lan F](https://www.ncbi.nlm.nih.gov/pubmed/?term=Lan%20F%5BAuthor%5D&cauthor=true&cauthor_uid=22514710), [Cacicedo JM](https://www.ncbi.nlm.nih.gov/pubmed/?term=Cacicedo%20JM%5BAuthor%5D&cauthor=true&cauthor_uid=22514710), [Chen TC](https://www.ncbi.nlm.nih.gov/pubmed/?term=Chen%20TC%5BAuthor%5D&cauthor=true&cauthor_uid=22514710), [Breton L](https://www.ncbi.nlm.nih.gov/pubmed/?term=Breton%20L%5BAuthor%5D&cauthor=true&cauthor_uid=22514710), [Ruderman NB](https://www.ncbi.nlm.nih.gov/pubmed/?term=Ruderman%20NB%5BAuthor%5D&cauthor=true&cauthor_uid=22514710).
53. [Int Immunopharmacol.](https://www.ncbi.nlm.nih.gov/pubmed/26774212) 2016 Mar;32:1-7. doi: 10.1016/j.intimp.2016.01.002. Resveratrol-induced autophagy promotes survival and attenuates doxorubicin-induced cardiotoxicity. [Gu J](https://www.ncbi.nlm.nih.gov/pubmed/?term=Gu%20J%5BAuthor%5D&cauthor=true&cauthor_uid=26774212), [Hu W](https://www.ncbi.nlm.nih.gov/pubmed/?term=Hu%20W%5BAuthor%5D&cauthor=true&cauthor_uid=26774212), [Song ZP](https://www.ncbi.nlm.nih.gov/pubmed/?term=Song%20ZP%5BAuthor%5D&cauthor=true&cauthor_uid=26774212), [Chen YG](https://www.ncbi.nlm.nih.gov/pubmed/?term=Chen%20YG%5BAuthor%5D&cauthor=true&cauthor_uid=26774212), [Zhang DD](https://www.ncbi.nlm.nih.gov/pubmed/?term=Zhang%20DD%5BAuthor%5D&cauthor=true&cauthor_uid=26774212), [Wang CQ](https://www.ncbi.nlm.nih.gov/pubmed/?term=Wang%20CQ%5BAuthor%5D&cauthor=true&cauthor_uid=26774212).
54. [J Biol Chem.](https://www.ncbi.nlm.nih.gov/pubmed/20080969) 2010 Mar 19;285(12):9100-13. doi: 10.1074/jbc.M109.060061. AMP-activated protein kinase signaling activation by resveratrol modulates amyloid-beta peptide metabolism. [Vingtdeux V](https://www.ncbi.nlm.nih.gov/pubmed/?term=Vingtdeux%20V%5BAuthor%5D&cauthor=true&cauthor_uid=20080969), [Giliberto L](https://www.ncbi.nlm.nih.gov/pubmed/?term=Giliberto%20L%5BAuthor%5D&cauthor=true&cauthor_uid=20080969), [Zhao H](https://www.ncbi.nlm.nih.gov/pubmed/?term=Zhao%20H%5BAuthor%5D&cauthor=true&cauthor_uid=20080969), [Chandakkar P](https://www.ncbi.nlm.nih.gov/pubmed/?term=Chandakkar%20P%5BAuthor%5D&cauthor=true&cauthor_uid=20080969), [Wu Q](https://www.ncbi.nlm.nih.gov/pubmed/?term=Wu%20Q%5BAuthor%5D&cauthor=true&cauthor_uid=20080969), [Simon JE](https://www.ncbi.nlm.nih.gov/pubmed/?term=Simon%20JE%5BAuthor%5D&cauthor=true&cauthor_uid=20080969), [Janle EM](https://www.ncbi.nlm.nih.gov/pubmed/?term=Janle%20EM%5BAuthor%5D&cauthor=true&cauthor_uid=20080969), [Lobo J](https://www.ncbi.nlm.nih.gov/pubmed/?term=Lobo%20J%5BAuthor%5D&cauthor=true&cauthor_uid=20080969), [Ferruzzi MG](https://www.ncbi.nlm.nih.gov/pubmed/?term=Ferruzzi%20MG%5BAuthor%5D&cauthor=true&cauthor_uid=20080969), [Davies P](https://www.ncbi.nlm.nih.gov/pubmed/?term=Davies%20P%5BAuthor%5D&cauthor=true&cauthor_uid=20080969), [Marambaud P](https://www.ncbi.nlm.nih.gov/pubmed/?term=Marambaud%20P%5BAuthor%5D&cauthor=true&cauthor_uid=20080969).
55. [BMC Cancer.](https://www.ncbi.nlm.nih.gov/pubmed/29788929) 2018 May 22;18(1):581. doi: 10.1186/s12885-018-4504-5. Resveratrol induces autophagy-dependent apoptosis in HL-60 cells. [Fan Y](https://www.ncbi.nlm.nih.gov/pubmed/?term=Fan%20Y%5BAuthor%5D&cauthor=true&cauthor_uid=29788929), [Chiu JF](https://www.ncbi.nlm.nih.gov/pubmed/?term=Chiu%20JF%5BAuthor%5D&cauthor=true&cauthor_uid=29788929), [Liu J](https://www.ncbi.nlm.nih.gov/pubmed/?term=Liu%20J%5BAuthor%5D&cauthor=true&cauthor_uid=29788929)^2^, [Deng Y](https://www.ncbi.nlm.nih.gov/pubmed/?term=Deng%20Y%5BAuthor%5D&cauthor=true&cauthor_uid=29788929), [Xu C](https://www.ncbi.nlm.nih.gov/pubmed/?term=Xu%20C%5BAuthor%5D&cauthor=true&cauthor_uid=29788929), [Zhang J](https://www.ncbi.nlm.nih.gov/pubmed/?term=Zhang%20J%5BAuthor%5D&cauthor=true&cauthor_uid=29788929), [Li G](https://www.ncbi.nlm.nih.gov/pubmed/?term=Li%20G%5BAuthor%5D&cauthor=true&cauthor_uid=29788929).
56. [Molecules.](https://www.ncbi.nlm.nih.gov/pubmed/30213073) 2018 Sep 12;23(9). pii: E2327. doi: 10.3390/molecules23092327. Resveratrol Suppresses Matrix Metalloproteinase-2 Activation Induced by Lipopolysaccharide in Mouse Osteoblasts via Interactions with AMP-Activated Protein Kinase and Suppressor of Cytokine Signaling 1. [Yu Y](https://www.ncbi.nlm.nih.gov/pubmed/?term=Yu%20Y%5BAuthor%5D&cauthor=true&cauthor_uid=30213073), [Li X](https://www.ncbi.nlm.nih.gov/pubmed/?term=Li%20X%5BAuthor%5D&cauthor=true&cauthor_uid=30213073), [Mi J](https://www.ncbi.nlm.nih.gov/pubmed/?term=Mi%20J%5BAuthor%5D&cauthor=true&cauthor_uid=30213073), [Qu L](https://www.ncbi.nlm.nih.gov/pubmed/?term=Qu%20L%5BAuthor%5D&cauthor=true&cauthor_uid=30213073), [Yang D](https://www.ncbi.nlm.nih.gov/pubmed/?term=Yang%20D%5BAuthor%5D&cauthor=true&cauthor_uid=30213073), [Guo J](https://www.ncbi.nlm.nih.gov/pubmed/?term=Guo%20J%5BAuthor%5D&cauthor=true&cauthor_uid=30213073), [Qiu L](https://www.ncbi.nlm.nih.gov/pubmed/?term=Qiu%20L%5BAuthor%5D&cauthor=true&cauthor_uid=30213073).
57. [J Cell Biochem.](https://www.ncbi.nlm.nih.gov/pubmed/29663499) 2018 Jul;119(7):6162-6172. doi: 10.1002/jcb.26822. Resveratrol inhibits the proliferation and induces the apoptosis in ovarian cancer cells via inhibiting glycolysis and targeting AMPK/mTOR signaling pathway. [Liu Y](https://www.ncbi.nlm.nih.gov/pubmed/?term=Liu%20Y%5BAuthor%5D&cauthor=true&cauthor_uid=29663499), [Tong L](https://www.ncbi.nlm.nih.gov/pubmed/?term=Tong%20L%5BAuthor%5D&cauthor=true&cauthor_uid=29663499), [Luo Y](https://www.ncbi.nlm.nih.gov/pubmed/?term=Luo%20Y%5BAuthor%5D&cauthor=true&cauthor_uid=29663499), [Li X](https://www.ncbi.nlm.nih.gov/pubmed/?term=Li%20X%5BAuthor%5D&cauthor=true&cauthor_uid=29663499), [Chen G](https://www.ncbi.nlm.nih.gov/pubmed/?term=Chen%20G%5BAuthor%5D&cauthor=true&cauthor_uid=29663499), [Wang Y](https://www.ncbi.nlm.nih.gov/pubmed/?term=Wang%20Y%5BAuthor%5D&cauthor=true&cauthor_uid=29663499).
58. [Autophagy.](https://www.ncbi.nlm.nih.gov/pubmed/27533078) 2016 Nov;12(11):2009-2025. Hypothalamic AMPK-induced autophagy increases food intake by regulating NPY and POMC expression. [Oh TS](https://www.ncbi.nlm.nih.gov/pubmed/?term=Oh%20TS%5BAuthor%5D&cauthor=true&cauthor_uid=27533078), [Cho H](https://www.ncbi.nlm.nih.gov/pubmed/?term=Cho%20H%5BAuthor%5D&cauthor=true&cauthor_uid=27533078), [Cho JH](https://www.ncbi.nlm.nih.gov/pubmed/?term=Cho%20JH%5BAuthor%5D&cauthor=true&cauthor_uid=27533078), [Yu SW](https://www.ncbi.nlm.nih.gov/pubmed/?term=Yu%20SW%5BAuthor%5D&cauthor=true&cauthor_uid=27533078), [Kim EK](https://www.ncbi.nlm.nih.gov/pubmed/?term=Kim%20EK%5BAuthor%5D&cauthor=true&cauthor_uid=27533078).
59. [Cell Rep.](https://www.ncbi.nlm.nih.gov/pubmed/30517873) 2018 Dec 4;25(10):2878-2890.e4. doi: 10.1016/j.celrep.2018.11.042. ULK1 O-GlcNAcylation Is Crucial for Activating VPS34 via ATG14L during Autophagy Initiation. [Pyo KE](https://www.ncbi.nlm.nih.gov/pubmed/?term=Pyo%20KE%5BAuthor%5D&cauthor=true&cauthor_uid=30517873), [Kim CR](https://www.ncbi.nlm.nih.gov/pubmed/?term=Kim%20CR%5BAuthor%5D&cauthor=true&cauthor_uid=30517873), [Lee M](https://www.ncbi.nlm.nih.gov/pubmed/?term=Lee%20M%5BAuthor%5D&cauthor=true&cauthor_uid=30517873), [Kim JS](https://www.ncbi.nlm.nih.gov/pubmed/?term=Kim%20JS%5BAuthor%5D&cauthor=true&cauthor_uid=30517873), [Kim KI](https://www.ncbi.nlm.nih.gov/pubmed/?term=Kim%20KI%5BAuthor%5D&cauthor=true&cauthor_uid=30517873), [Baek SH](https://www.ncbi.nlm.nih.gov/pubmed/?term=Baek%20SH%5BAuthor%5D&cauthor=true&cauthor_uid=30517873).
60. [Cell Signal.](https://www.ncbi.nlm.nih.gov/pubmed/23000343) 2013 Jan;25(1):50-65. doi: 10.1016/j.cellsig.2012.09.020. Starvation-induced autophagy is regulated by mitochondrial reactive oxygen species leading to AMPK activation. [Li L](https://www.ncbi.nlm.nih.gov/pubmed/?term=Li%20L%5BAuthor%5D&cauthor=true&cauthor_uid=23000343), [Chen Y](https://www.ncbi.nlm.nih.gov/pubmed/?term=Chen%20Y%5BAuthor%5D&cauthor=true&cauthor_uid=23000343), [Gibson SB](https://www.ncbi.nlm.nih.gov/pubmed/?term=Gibson%20SB%5BAuthor%5D&cauthor=true&cauthor_uid=23000343).
61. [Am J Physiol Cell Physiol.](https://www.ncbi.nlm.nih.gov/pubmed/20810907) 2010 Nov;299(5):C1171-9. doi: 10.1152/ajpcell.00514.2009. A role for AMPK in increased insulin action after serum starvation. [Ching JK](https://www.ncbi.nlm.nih.gov/pubmed/?term=Ching%20JK%5BAuthor%5D&cauthor=true&cauthor_uid=20810907), [Rajguru P](https://www.ncbi.nlm.nih.gov/pubmed/?term=Rajguru%20P%5BAuthor%5D&cauthor=true&cauthor_uid=20810907), [Marupudi N](https://www.ncbi.nlm.nih.gov/pubmed/?term=Marupudi%20N%5BAuthor%5D&cauthor=true&cauthor_uid=20810907), [Banerjee S](https://www.ncbi.nlm.nih.gov/pubmed/?term=Banerjee%20S%5BAuthor%5D&cauthor=true&cauthor_uid=20810907), [Fisher JS](https://www.ncbi.nlm.nih.gov/pubmed/?term=Fisher%20JS%5BAuthor%5D&cauthor=true&cauthor_uid=20810907).
